# Epigenome-Wide Tobacco-Related Methylation Signature Identification and Their Multilevel Regulatory Network Inference for Lung Adenocarcinoma

**DOI:** 10.1155/2020/2471915

**Published:** 2020-04-24

**Authors:** Yan-Mei Dong, Ming Li, Qi-En He, Yi-Fan Tong, Hong-Zhi Gao, Yi-Zhi Zhang, Ya-Meng Wu, Jun Hu, Ning Zhang, Kai Song

**Affiliations:** ^1^School of Chemical Engineering and Technology, Tianjin University, 300072 Tianjin, China; ^2^Department of Biomedical Engineering, Tianjin Key Lab of Biomedical Engineering Measurement, Tianjin University, 300072 Tianjin, China; ^3^The Second Affiliated Hospital of Fujian Medical University, 362000 Quanzhou, Fujian, China; ^4^Department of Hematology and Oncology, Karamay Central Hospital, 834000 Karamay, Xinjiang Uygur Autonomous Region, China

## Abstract

Tobacco exposure is one of the major risks for the initiation and progress of lung cancer. The exact corresponding mechanisms, however, are mainly unknown. Recently, a growing body of evidence has been collected supporting the involvement of DNA methylation in the regulation of gene expression in cancer cells. The identification of tobacco-related signature methylation probes and the analysis of their regulatory networks at different molecular levels may be of a great help for understanding tobacco-related tumorigenesis. Three independent lung adenocarcinoma (LUAD) datasets were used to train and validate the tobacco exposure pattern classification model. A deep selecting method was proposed and used to identify methylation signature probes from hundreds of thousands of the whole epigenome probes. Then, BIMC (biweight midcorrelation coefficient) algorithm, SRC (Spearman's rank correlation) analysis, and shortest path tracing method were explored to identify associated genes at gene regulation level and protein-protein interaction level, respectively. Afterwards, the KEGG (Kyoto Encyclopedia of Genes and Genomes) pathway analysis and GO (Gene Ontology) enrichment analysis were used to analyze their molecular functions and associated pathways. 105 probes were identified as tobacco-related DNA methylation signatures. They belong to 95 genes which are involved in hsa04512, hsa04151, and other important pathways. At gene regulation level, 33 genes are uncovered to be highly related to signature probes by both BIMC and SRC methods. Among them, FARSB and other eight genes were uncovered as Hub genes in the gene regulatory network. Meanwhile, the PPI network about these 33 genes showed that MAGOH, FYN, and other five genes were the most connected core genes among them. These analysis results may provide clues for a clear biological interpretation in the molecular mechanism of tumorigenesis. Moreover, the identified signature probes may serve as potential drug targets for the precision medicine of LUAD.

## 1. Introduction

Lung cancer has been the leading cause of cancer-related mortality throughout the world for decades [1]. It has been shown that smokers 15–30 times more likely to get lung cancer or die from it than lifetime never-smokers. Moreover, smoking during cancer therapy may influence radiotherapy and chemotherapy outcomes [2]. Tobacco exposure is the major risk for lung cancer; however, 10% to 15% of patients still have no history of it [3, 4]. Being the major form of lung cancer associated with never-smokers [5–7], when considering therapies for lung adenocarcinoma (LUAD) patients, the carcinogenic mechanisms of smokers are believed to differ from those of never-smokers. The rising trend in the proportion of never-smokers in LUAD urgently requires the understanding of such differences on molecular levels for the development of precision medicine.

DNA methylation, a useful and stable surrogate of the genetic response, has recently been suggested to be one of the potential mechanisms for smoking-related health outcomes [8–11]. Many studies have demonstrated that smoking can induce genomic instability by producing genetic mutations and altering epigenetic modifications. Recently, epigenome-wide association studies (EWAS) have opened a new avenue for lung cancer research. Hypermethylation of promoter regions has frequently been observed in smokers with and without lung cancer [12]. The cumulative smoking dose (pack-years) has been shown to correlate well with the frequency of methylation in cancer-free heavy smokers [13]. However, the highly reproducible DNA methylation markers linked to smoking and their corresponding mechanism remain unclear.

**Figure 1:**
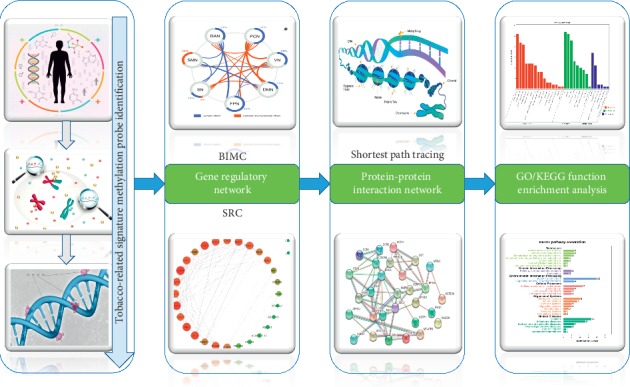
Flowchart of current research. ^*∗*^Cited from “Genetic and Environmental Contributions to Functional Connectivity Architecture of the Human Brain.”

In our previous study, we identified 48 genes whose promoter DNA methylation levels were highly related to tobacco exposure for LUAD patients. In that paper, the average methylation value of the probes located in a gene's promoter area was used as the methylation value of that gene. Then, such average methylation values of protein-coding genes were used as variables to do the tobacco exposure classification. In short, our previous study was limited in analyzing promoter methylation levels of protein-coding genes. Recent studies, however, show that DNA methylation in the body areas of genes might be involved in differential promoter usage and also in transcription elongation and alternative splicing [14–16]. Gene body methylation has long been ignored but is more prevalent in the genome than promoter hypermethylation. It may be associated with increased gene expression. Therefore, investigating every probe as an individual variable rather than investigating only promoter probes of protein-coding genes may uncover real signature markers. Correspondingly, the aim of this study is twofold: (1) to identify signature methylation probes highly related to tobacco exposure from hundreds of thousands of epigenome-wide probes and (2) to uncover the important genes related to these signature probes from different molecular levels, i.e., gene regulatory level and protein-protein interaction level.  Figure 1 and Figure S1 show the corresponding flowchart of our study.

## 2. Materials and Methods

### 2.1. Data

Two GEO (Gene Expression Omnibus) datasets and one TCGA (The Cancer Genome Atlas) dataset were used for training and validation, respectively. They are summarized in Table 1.

These two GEO datasets with access numbers of GSE39279 and GSE66836 were downloaded from https://www.ncbi.nlm.nih.gov/geo/ [17]. GSE39279 was used as the training data, while GSE66836 was used as a validation data. Their methylation levels (*β*-value) of 485,577 CpG-probes were collected via the Infinium Human Methylation 450 BeadChip. Their clinical information was collected from the supplemental document of reference [18].

TCGA dataset was downloaded through the public Genomic Data Commons Data Portal (GDC: https://portal.gdc.cancer.gov/). The level 3 methylation data of LUAD patients measured by Infinium Human Methylation 450 BeadChip was used as the second validation sample set. Additionally, the level 3 mRNA expression data measured by the Illumina HiSeq 2000 RNA Sequencing Version 2 platform was also downloaded and used in our study.

For LUAD patients, we shall use the following definitions for tobacco usage and exposure [19]:

- Current smoker: an adult who has smoked at least 100 cigarettes in their lifetime and who currently smokes cigarettes or has quit within the previous 12 months.
- Never smoker: an adult who has never smoked or has smoked less than 100 cigarettes in their lifetime.
- Ever smoker: an adult who has smoked at least 100 cigarettes in their lifetime (irrespective of whether they are currently smoking).

### 2.2. Statistic Methods

#### 2.2.1. Jarque–Bera Test

In statistics, the Jarque–Bera test is a goodness-of-fit test to decide whether sample data have the skewness and kurtosis matching a normal distribution [20].

Due to the unknown distribution of the probes, Jarque–Bera test was used to test their goodness-of-fit of the normal distribution.

#### 2.2.2. Wilcoxon Rank-Sum Test

The Wilcoxon rank-sum test (also called the Mann–Whitney-Wilcoxon (MWW), Mann–Whitney *U* test, or Wilcoxon-Mann–Whitney test) is a nonparametric test determining whether two independent samples were selected from populations having the same distribution.

Even though the *t*-test is one of the most widely used statistical procedures, but it assumes that the variable in question is normally distributed in the two groups. When this assumption is in doubt, the nonparametric Wilcoxon rank-sum test is suggested as an alternative to the *t*-test, which does not rely on the distributional assumptions [21].

Wilcoxon rank-sum test with Benjamini-Hochberg correction, a powerful tool to control the false discovery rate (FDR), was used to select probes whose patterns are significantly different between current-smokers and never-smokers.

#### 2.2.3. Partial Least Squares (PLS)

PLS [22] is a widely used algorithm for modeling the relationship between sets of observed variables by means of latent variables. It comprises regression and classification tasks as well as dimension reduction and modeling [23, 24]. Instead of finding hyperplanes of minimum variance between the response and independent variables, it finds a linear regression model by projecting the predicted variables (i.e., classification labels) and the observed variables (methylation values of probes in our case) to a new lower space [25–27]. Therefore, it performs very well for the analysis of high-dimension-small-sample data in bioinformatics.

#### 2.2.4. Biweight Midcorrelation Coefficient (BIMC) Algorithm

BIMC (biweight midcorrelation coefficient) algorithm was proposed by Yuan et al. [28]. Pearson's correlation coefficient is a representative method of correlation coefficient approaches. However, Pearson's correlation coefficient is sensitive to outliers. Biweight midcorrelation is considered to be a good alternative to Pearson's correlation coefficient since it is more robust to outliers.

### 2.3. Spearman's Rank Correlation

Unlike Pearson's correlation coefficient, Spearman's correlation coefficient is more accurate in describing the correlation in the nonlinear relationship and is not sensitive to significant outliers in samples.

#### 2.3.1. Differential Correlation Strategy

After calculating the correlation coefficients using BIMC or SRC [28], the Differential Correlation Strategy (DCS) was used to identify genes that are significantly associated with the identified signature probes throughout the whole genome genes. To do this, the genome-wide expression (GE) data and the epigenome-wide methylation (ME) data were used together as the input variables to BIMC or SRC to calculate their correlation coefficients. Afterwards, the correlation coefficients were used as input variables to the DCS algorithm. In this study, the two thresholds TS1 and TS2 of DCS were optimized as 0.3 and 0.6, respectively.

### 2.4. Data Preprocessing

For all datasets, genes whose GE or ME values were missing in all samples were removed. The GE data was log 2 transformed. Patient samples lacking any of the important clinical parameters (age, gender, cancer stage, overall survival time, and vital status) were removed. Genes and probes on X and Y chromosomes were removed. As a result, 16050 genes remained in GE data and 374176 probes were available in ME data, respectively.

### 2.5. Signature Probe Identification

The big challenge in bioinformatics, especially in epigenomic analysis, is the tremendous overwhelming imbalance between the variable size and the sample size. In our case, there were several thousands of probes but less than 180 samples in each dataset. The number of variables was more than 20 times the number of samples. It is not possible to identify signature probes in one step. Therefore, a new deep selecting method was proposed to identify tobacco-related signature probes.

Learning from the concept of “Deep Learning” methods for extracting features step by step, the proposed deep selecting method based on PLS extracts features and removes the least important variables step by step. It consists of the following steps: (1) using Wilcoxon rank-sum test to roughly select probes whose methylation levels were significantly different between smokers and never-smokers; (2) initiating a tobacco exposure pattern classification model with methylation values of all significantly different probes as input variables; (3) sorting probes according to their contributions to the classification model; (4) removing the least important probes; (5) remodeling the classification model; (6) repeating steps 3–6 iteratively until the classification accuracy could not be improved any more. Then, the remaining probes are considered as the signature probes since using only their ME values can accurately predict the tobacco exposure pattern of patients. Figure S1 shows the corresponding pipeline of it.

Fivefold cross-validation was explored to train the classification model. The sensitivity (SN), specificity (SP), and accuracy (ACC) were used to evaluate the classification performance of the model. Please see the supplementary document for their definitions.

### 2.6. Uncovering Related Genes at the Gene Regulatory Level

To identify genes significantly related to our identified signature probes, BIMC and SRC integrated into the DCS methods were explored with the GE and ME values of the whole genome-wide genes as input variables, respectively. Their corresponding gene regulatory network was visualized using OmicShare, a free online platform for data analysis (http://www.omicshare.com/).

### 2.7. Uncovering Related Genes at the Protein-Protein Interaction Level

The betweenness of a shortest path protein is the number of shortest paths across the protein. Then, the shortest path proteins were ranked by betweenness in descending order.

The proteins whose betweenness was greater than 3,000 were picked out and their corresponding genes were treated as genes related to our identified signature probes at the PPI level. The Dijkstra algorithm served to find the shortest path in the graph G between two given proteins, which was implemented in the R package “igraph” [29]. In order to ensure the validity and precision of our results, we randomly chose 134 proteins in the PPI network for the shortest path tracing and repeated the procedure 100 times, and a permutation test was performed. Then, we removed 5 genes that appear more frequently in randomized results, which were TP53, AKT1, HSP90AA, CTNNB1, and UBC [30, 31].

A weighted PPI network [32] among related genes was obtained from the STRING (Search Tool for the Retrieval of Interacting Genes) database (version 10.0). The STRING database is a database for searching known and predicted interactions between proteins (http://string.embl.de/) [33]. The ID of the human species is 9,606. There are 8,548,002 pairs of related interaction forces in it. The R package “STRINGdb” was used to map the corresponding protein IDs of the identified signature genes. Moreover, in this network, proteins are denoted as nodes and the interaction of every two proteins is given as an edge marked with a *d* score. The *d* value can be considered to represent the protein distances to each other: a smaller distance value indicates that the protein pair has a higher interaction confidence score, which means they may have more analogous functions.

### 2.8. Genes and Genomes Pathway Analysis and Gene Ontology Enrichment Analysis

KEGG (Kyoto Encyclopedia of Genes and Genomes) is a database resource for understanding high-level functions and utilities of biological systems, such as cells, organisms, and ecosystems, from molecular-level information. Such information is obtained from large-scale molecular datasets generated by genome sequencing and other high-throughput experimental technologies [34]. To do KEGG pathway analysis, the obtained differential gene identifiers are transformed into Ensemble ID by DAVID online analysis tool (https://david.ncifcrf.gov/) [35]. KEGG pathway analysis and GO (Gene Ontology) enrichment analysis were performed to analyze the functions of genes to which the signature probes belong. To do this, the KOBAS (http://kobas.cbi.pku.edu.cn/) online analyzing tool was used [36–38].

The flowchart of tobacco exposure pattern signature probe identification and their multilevel regulatory network analysis is shown in Figure 1 and Figure S1. All analyses were performed using MATLAB or R codes. Please refer to supplementary materials for more details.

## 3. Results

### 3.1. Identified Signature Probes and Their Classification Performance

According to the result obtained by the Jarque–Bera test, only 16.08% of 374176 probes were normally distributed. Therefore, the nonparametric Wilcoxon rank-sum test with the Benjamini-Hochberg FDR was used to roughly select probes whose methylation patterns in smokers and never-smokers were significantly different. As a result, 3,557 probes were selected at this step.

Then, the proposed deep selecting method based on PLS was used to identify the real signature probes from these 3557 probes. Considering the prediction accuracies of all training and validation datasets, 105 probes were eventually identified as signatures because using only their methylation values can achieve the best classification performance.  Table 2 lists their probe IDs and the corresponding genes. Other details are listed in Table S1 in the supplementary materials including their median methylation values in smoking and nonsmoking groups and their *P* values (Wilcoxon rank-sum test) between these two groups, respectively.

These 105 probes are located in different regions of 95 genes. For clarity, they were called signature genes. Six signature genes were related to multiple probes. They were AHRR (cg05575921, cg26076054, and cg00976097), ANKRD45 (cg13990746 and cg15680620), CYP1B1 (cg09799983 and cg11751707), PTPRN2 (cg09194449, cg10650290, and cg14419740), SP5 (cg07813142 and cg24772753), and SYT2 (cg05752786 and cg16315376).

Twenty signature probes are not located in any genes, which means these signature probes may locate at intragenic areas or their information remains unknown due to the experimental limitations.

The corresponding classification performance is shown in Table 3. The sensitivity, specificity, and accuracy of the training set are 98.04%, 100%, and 98.62%, respectively. All sensitivities, specificities, and accuracies of our two independent validation sets are higher than 80%. Moreover, the differences between these sensitivities and specificities are smaller than 4.2%. All these results indicated that the reliability of the model is very high. They also confirmed the essentiality of the identified signature probes.

**Figure 2:**
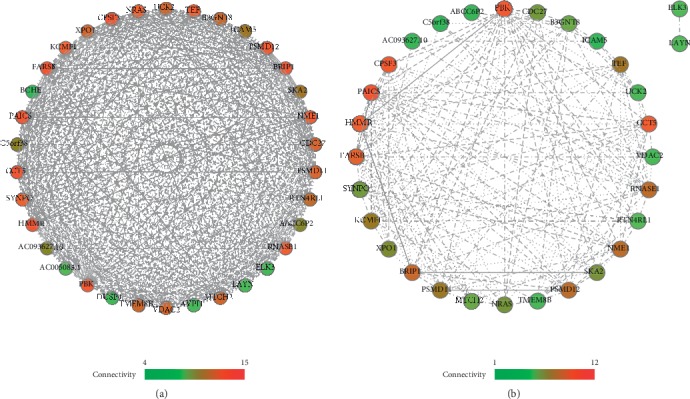
The gene weight network of important genes. The soft threshold was used to calculate the connectivity of the genes. Gene connectivity was expressed using the color of nodes (nodes representing genes). (a) A gene weight network constructed by 34 genes. (b) A gene weight network of part of the genes with significant weights performed a map of gene weights.

To uncover the genes strongly related to our identified signature probes or signature genes, both BIMC and SRC methods were ted into the DCS method to analyze the whole genome genes using their GE and ME values. Using the BIMC method, 84 genes were uncovered to be highly correlated with signature probes. Meanwhile, using the SRC method, 134 genes were uncovered to be highly related to signature probes. Among them, 34 genes were in common.  Figure 2 shows the gene regulatory network of these 34 genes. In this network, FARSB, PAICS, HMMR, PBK, NRAS, BRIP1, PSMD12, PSMD11, and SKA2 have the highest connections (>10.75), which means they are the core genes in this regulatory network. For clarity, they were named 84 BIMC genes, 134 SRC genes, and 34 common genes.

**Figure 3:**
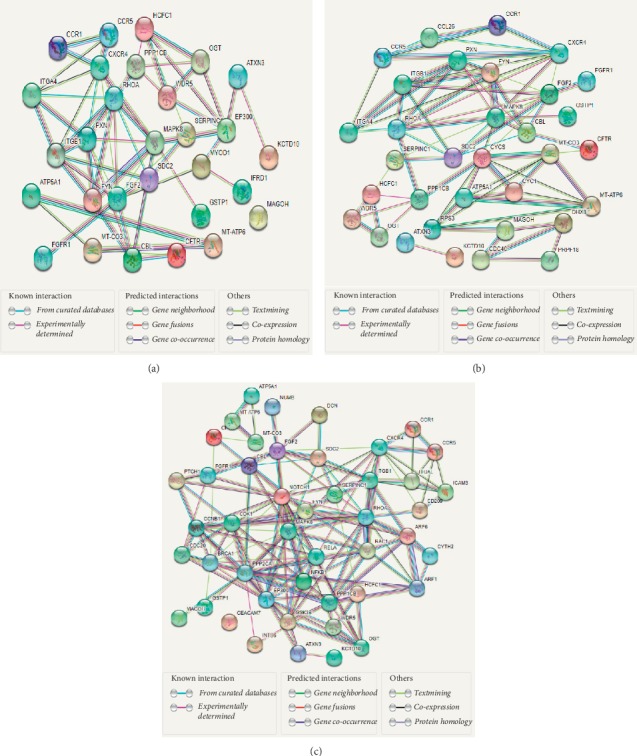
The protein-protein interaction (PPI) networks. (a) PPI corresponding to the 95 signature genes (the genes corresponding to the protein whose betweenness is greater than or equal to 100). (b) PPI corresponding to the 84 BIMC genes (the genes corresponding to the protein whose betweenness is greater than or equal to 100). (c) PPI corresponding to the 134 SRC genes (the genes corresponding to the protein whose betweenness is greater than or equal to 200).

Protein regulation analysis was performed on 95 signature genes, 84 BIMC genes, and 134 SRC genes using the shortest path tracing algorithm. The corresponding protein-protein interaction (PPI) networks were shown in Figure 3 with dots representing proteins and edges representing interactions between proteins.

### 3.2. Molecular Function and Pathway Analysis

The significant biological functions of 95 signature genes obtained by KEGG pathway analysis and GO analysis are listed in Tables S2 and S3 in the supplementary materials. Nine pathways were significantly enriched.

Three of them may be very important for LUAD:

- *hsa04512*. Specific interactions between cells and the ECM are mediated by transmembrane molecules, mainly integrins and perhaps also proteoglycans, CD36, or other cell-surface-associated components. These interactions lead to a direct or indirect control of cellular activities such as adhesion, migration, differentiation, proliferation, and apoptosis.
- *hsa04151*. The phosphatidylinositol 3′-kinase- (PI3K-) Akt signaling pathway is activated by many types of cellular stimuli or toxic insults and regulates fundamental cellular functions such as transcription, translation, proliferation, growth, and survival.
- *hsa04510*. Cell-matrix adhesions play essential roles in important biological processes including cell motility, cell proliferation, cell differentiation, regulation of gene expression, and cell survival.

## 4. Discussion

As we mentioned above, identifying signature probes throughout hundreds of thousands of probes was a big challenge in our case. Therefore, we proposed a deep selecting method to overcome it. Least important probes were supposed to have more noise than useful information. Removing them can improve the classification accuracy. In every round of deep selecting method, the comparative contributions significantly varied after removing noisy probes. Therefore, the remodeling of the classification model and recalculating of the contributions of probes in steps 3–5 of this method were very important.

With this method, 105 probes were identified as tobacco-related signatures. Using only their methylation values, the tobacco exposure patterns of LUAD patients can be classified correctly. For the training dataset GSE39279, the accuracy of the 105-signature probe classifier was 98.62%. Such a high accuracy usually indicates the overfitting of the model training. But the accuracies of two independent validation datasets were both higher than 83%, which proves the high quality in model generalization. More importantly, these two validation datasets (GSE66836 and TCGA) were collected by two different organizations from two different LUAD cohorts. The good-enough accuracies for both of them strongly proved the satisfactory predictive performance of the classification model. It also strongly proved the essentiality of the identified signature probes.

**Figure 4:**
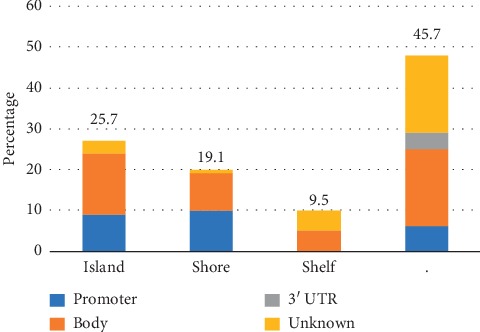
Distribution of feature types of 105 signature probes.

Figure 4 shows that the 105 probes were mainly located in the open sea (45.7%) and island (25.7%) regions. On the contrary, the percentages of probes distributed over shore (19.1%) and shelf (9.5%) areas were very low. Additionally, these probes were mainly distributed in the promoter region (23.8%) and the body region (45.7%).

Apart from six signature genes being related to multiple probes, there are also 16 probes belonging to multiple genes. Normally, for one-probe-multiple-gene cases, the first gene in the list is the most likely one corresponding to that probe. Therefore, the first gene was chosen as the corresponding gene to that probe. Therefore, for multiple-gene cases, we chose the probe and the first corresponding gene in the list for further relationship analysis.  Table S4 lists the coefficient values between 85 probes and 77 genes (multiple-probe-one-gene is allowed). Among these 85 probes, 25 of them are located in the promoter area. But only 15 of these 25 promoter probes negatively regulate their genes. The methylations of the other 10 have positive relationships with their genes' expression values. For signature probes located at the body area of genes, 28 of them negatively regulate their gene expression while the other 20 positively regulate their gene expression.

In addition, Figure S2 shows the Volcano plot of the expression levels of 95 genes corresponding to 105 probes in smoker and never-smoker groups. The detailed information is shown in Table S5 in the supplementary materials. It can be seen from Table S5 that smoking alters the level of gene expression. ECM1, AHRR, GPR15, and PR11-351M16.3 were significantly upregulated in smokers. On the contrary, LINC01019 and MFSD4 were significantly downregulated in never-smokers.

From the literature research using PubMed, 25 of the 95 signature genes are associated with smoking, 6 are associated with LUAD, 2 are associated with LUSC, 4 are associated with NSCLC, 9 are associated with lung cancer, 22 are associated with cancer, and the remaining 34 genes are still unclear (see Table S6).

**Figure 5:**
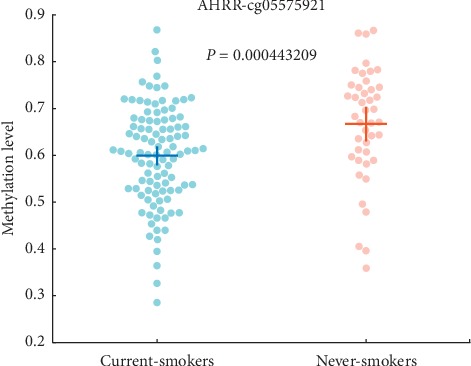
Beeswarm plot of gene AHRR (cg05575921).

Among these 95 signature genes, AHRR is one of the most important genes. It corresponds to cg00976097, cg05575921, and cg26076054 probes in 105 signature probes. Several published reports showed: (1) the DNA methylation of it in lung tissue of smokers was significantly lower than that in nonsmokers, while DNA methylation was inversely proportional to the level of AHRR mRNA in smokers and nonsmokers [39, 40]; (2) the protein encoded by AHRR is involved in the regulation of cell growth and differentiation and the immune system [41]; (3) AHRR was found to be the most significantly different probe set between never-smokers and current-smokers [41–43].  Figure 5 shows the methylation levels of cg05575921 in current-smoker and never-smoker samples. The *P* values were 0.4*E* – 4 which means that its methylation levels were significantly different in these two group samples.

Figure 2 shows the gene regulatory network of related genes uncovered at the gene regulatory level. FARSB, PAICS, HMMR, PBK, NRAS, BRIP1, PSMD12, PSMD11, and SKA2 were the most important genes because they had the most connections with other genes. Several of them have been reported to be important for LUAD or NSCLC as in the following examples:

- It is known from The Human Protein Atlas that FARSB is a protein-coding gene and a prognostic indicator for certain cancers [44].
- PAICS was pointed out to be used as a prognostic biomarker for aggressive LUAD [45].
- HMMR is closely related to the tumor outgrowth to the metastatic relapse of LUAD [46].
- Studies have shown that the expression of PBK/TOPK was significantly associated with the adverse prognosis in NSCLC [47].
- The NRAS gene provides instructions for making a protein called N-Ras that is involved primarily in regulating cell division. Ohashi et al. pointed out that NRAS mutations were common in people with a history of smoking in LUAD [48].
- Mutations in BRIP1 were frequently observed in LUAD [49].
- Both PSMD12 and PSMD11 are involved in cell cycle progression, apoptosis, or DNA damage repair [50].
- PRR11-SKA2 gene pair is shown to be synergistically overexpressed in lung cancer and participates in the process of lung cancer development [51].

Under the premise that the corresponding protein betweenness of the gene is greater than or equal to 100, we found that the genes obtained by the BIMC gene and the PPI network were a subset of the genes obtained by the SRC gene and the PPI network. Therefore, we only analyzed the genes obtained from the BIMC gene and their PPI network (see Table S7). In total, 33 possible related genes were uncovered. The top 8 genes with the highest betweenness were MAGOH, FYN, CBL, RHOA, MAPK8, CYC1, ITGB1, and CCL26.

- FYN is a member of the protein-tyrosine kinase oncogene family. It encodes a membrane-associated tyrosine kinase that has been implicated in the control of cell growth.
- Du et al. [52] determined that CBL plays a key role in the development and metastasis of lung tumors and that c-CBL mutations may contribute to the carcinogenic potential of MET and EGFR in lung cancer.
- RHOA is a protein-coding gene, and RHOA-related diseases include adenocarcinoma and peripheral T-cell lymphoma. Konstantinidou G et al. [53] revealed that RHOA is involved in the biological process of maintaining the signal axis required for KRAS-driven lung adenocarcinoma.
- The signaling pathway of MAPK8 affects the regulation of gene expression in non-small-cell lung cancer [54].
- Li et al. [55] stated clearly that CYC1 silencing inhibited growth in osteosarcoma cells via increasing apoptosis and damaging energy metabolism.
- Pellinen et al. [56] pointed out that ITGB1 is associated with poor prognosis of prostate cancer.
- CCL26 is involved in the regulation of eosinophil function in asthmatic patients [57].

Although these genes may not be indispensable markers associated with smoking in lung adenocarcinoma, they reveal that cancer usually has a common molecular mechanism and specific genes with type-dependent patterns.

## 5. Conclusions

The identification of biomolecular markers is one of the most important issues in biomedicine and genomics. Lung adenocarcinoma is a disease with a high mortality rate in cancer. Researchers are eager to find its related genes, which is helpful to expose its mechanism and to develop effective treatments. This study used statistical methods to find 105 signature probes, which are methylation biomarkers of lung adenocarcinoma associated with tobacco exposure patterns. The analysis of gene weighting regulatory networks and protein regulatory networks revealed genes that are directly or indirectly related to these 105 signature probes at different levels. It is hopeful that this research will be of great help to medical workers.

## Figures and Tables

**Table 1:** The summary of the clinical information of LUAD samples in all datasets.

LUAD	Discovery cohort	Validation cohort	
	GSE39279	TCGA	GSE66836
Number of samples	145	172	121
Age	40–90	33–84^*∗*^	39–84
Gender			
Females	76	91	66
Males	69	81	55
Smoking history			
Current	102	105	105
Never	43	67	16
Stage			
Stage I	84	89	70
Stage II	24	49	24
Stage III	30	23	25
Stage IV	7	10	2
Not applicable		1	
Smoking pack-years			
0–50	99	58	Unknown
51–100	35	19	Unknown
101–125	2	8	Unknown
Not applicable	9	87	Unknown

^*∗*^The ages of 4 smokers and 5 nonsmokers in TCGA data are unknown.

**Table 2:** List of 105 signature methylation probes.

Number	Probe	Chromosome	Gene symbol	Feature type
1	cg00334821	chr7	LIMK1	—
2	cg00370022	chr15	CYP1A1	N_Shelf
3	cg00688979	chr6	CCHCR1	—
4	cg00702638	chr3	KIAA1143; KIF15	Island
5	cg00976097	chr5	AHRR	Island
6	cg00993400	chr1	AL672294.1; ZNF692	S_Shore
7	cg01049916	chr10	FGFR2	—
8	cg01637537	chr22	FAM83F	N_Shore
9	cg01971034	chr1	—	N_Shelf
10	cg02050426	chr18	CDH20	Island
11	cg02387679	chr5	IQGAP2	Island
12	cg02498206	chr3	BOC	—
13	cg02826525	chr2	ARHGEF33; RP11-173C1.1	Island
14	cg02988118	chr2	—	N_Shelf
15	cg03078488	chr7	IGF2BP3	N_Shore
16	cg03277049	chr3	LINC00886	Island
17	cg03642695	chr17	TEKT3	—
18	cg03789372	chr8	—	—
19	cg03806812	chr2	—	—
20	cg03945895	chr1	PRDM2	—
21	cg03985801	chr1	LGR6	N_Shore
22	cg04267214	chr1	—	Island
23	cg04616529	chr16	CLEC16A	—
24	cg04865290	chr3	TMEM110; TMEM110-MUSTN1	N_Shelf
25	cg05033369	chr1	FCRLA	—
26	cg05559381	chr5	—	—
27	cg05575921	chr5	AHRR	N_Shore
28	cg05752786	chr1	SYT2	Island
29	cg05787209	chr16	STX1B	S_Shore
30	cg05951221	chr2	ECEL1P1	Island
31	cg06010163	chr6	—	—
32	cg06227763	chr18	—	—
33	cg06540950	chr5	—	—
34	cg06637330	chr5	—	N_Shelf
35	cg07160783	chr16	—	—
36	cg07325233	chr21	AP000295.9; IL10RB; IL10RB-AS1	Island
37	cg07709148	chr8	RP11-486M23.1	—
38	cg07813142	chr2	SP5	Island
39	cg08008475	chr13	RNY1P1	—
40	cg08374798	chr20	COL9A3	N_Shore
41	cg08733957	chr1	GALE	N_Shore
42	cg08894131	chr1	GJA5	—
43	cg09194449	chr7	PTPRN2	Island
44	cg09278187	chr1	FOXJ3	—
45	cg09370982	chr16	RP11-20I23.1; TBC1D24	S_Shore
46	cg09799983	chr2	CYP1B1; CYP1B1-AS1	Island
47	cg10076730	chr13	COL4A2	N_Shore
48	cg10354195	chr10	LRRC27	—
49	cg10385390	chr1	PARK7	S_Shore
50	cg10413224	chr7	BMPER	Island
51	cg10650290	chr7	PTPRN2	—
52	cg11545521	chr11	PTPRJ	—
53	cg11751707	chr2	CYP1B1; CYP1B1-AS1	Island
54	cg11954332	chr1	PRRX1	—
55	cg12020590	chr11	—	—
56	cg12387247	chr19	FCER2	—
57	cg13563863	chr19	FZR1	Island
58	cg13654445	chr9	NTRK2	—
59	cg13990746	chr1	ANKRD45	Island
60	cg14270346	chr9	RP11-613M10.9; SHB	—
61	cg14320852	chr9	—	—
62	cg14373988	chr1	PEX10	N_Shore
63	cg14419740	chr7	PTPRN2	Island
64	cg15233380	chr13	SHISA2	—
65	cg15513657	chr14	MEG3	—
66	cg15585555	chr1	RASSF5	S_Shore
67	cg15680620	chr1	ANKRD45	S_Shore
68	cg15922705	chr6	COL9A1	N_Shore
69	cg16315376	chr1	SYT2	Island
70	cg16322479	chr5	EXOC3; EXOC3-AS1	S_Shore
71	cg16377959	chr5	LINC01019	—
72	cg16840978	chr8	RAB11FIP1	N_Shelf
73	cg16884847	chr2	PRKCE	—
74	cg17211612	chr12	DNAH10	—
75	cg17373442	chr3	CHST2	Island
76	cg17676618	chr10	—	—
77	cg18001059	chr7	MRPL32; PSMA2	Island
78	cg18713316	chr1	KCNN3	Island
79	cg18883807	chr14	—	—
80	cg18919659	chr22	PACSIN2	—
81	cg19341901	chr7	—	—
82	cg19859270	chr3	CPOX; GPR15	—
83	cg20439473	chr17	VEZF1	N_Shelf
84	cg20459495	chr2	—	—
85	cg20538211	chr4	IGFBP7-AS1	N_Shelf
86	cg20546279	chr7	—	S_Shore
87	cg20628376	chr10	RP11-351M16.3	—
88	cg21012061	chr1	ELK4; MFSD4	—
89	cg21083936	chr11	—	S_Shelf
90	cg21500300	chr12	BCAT1; RP11-662I13.2	S_Shore
91	cg21885107	chr14	PAPLN; RP4-647C14.2	Island
92	cg23369748	chr6	SASH1	—
93	cg23501962	chr11	RP4-683L5.1; SLC1A2	N_Shore
94	cg23854567	chr12	PXN	—
95	cg24203542	chr11	NAV2	—
96	cg24279017	chr12	ETV6	—
97	cg24772753	chr2	SP5	Island
98	cg25192619	chr6	CCDC167	Island
99	cg26005485	chr8	FAM135B	N_Shore
100	cg26029292	chr8	ZNF7	S_Shelf
101	cg26076054	chr5	AHRR	Island
102	cg26582784	chr7	AC002454.1; CDK6	Island
103	cg26799398	chr1	ECM1; TARS2	—
104	cg26972614	chr11	IL18BP	—
105	cg27052537	chr7	—	—

**Table 3:** The classification performance obtained by the methylation values of 105 identified signature probes.

Data	Database	SN	SP	ACC
Training cohort	GSE39279	0.9804	1	0.9862
Validation cohort	TCGA	0.8476	0.806	0.8314
	GSE66836	0.8381	0.8125	0.8347

The strongly related genes and their regulatory network at different levels.

## Data Availability

The datasets analyzed during the current study are available at the GEO (https://www.ncbi.nlm.nih.gov/geo/) and GDC Data Portal (https://portal.gdc.cancer.gov/).

## Supplementary Materials

Supplementary Materials Figure S1: flow chart for the identification and classification of methylation biomarkers associated with smoking in lung adenocarcinoma. Figure S2: volcano plot of 95 signature genes in never-smokers versus smokers. Table S1: list of 105 signature methylation probes and their *P* values. Table S2: significantly enriched pathways of genes corresponding to 105 differential probes. Table S3: GO enrichment analysis of genes corresponding to 105 differential probes. Table S4: the relationship between methylation and expression. Table S5: the expression of 95 genes corresponding to 105 probes (current-smoker versus never-smoker). Table S6: related references and related types of 95 genes. Table S7: the 33 possible related genes corresponding to proteins whose betweenness is greater than or equal to 100 obtained by BIMC method using the shortest path algorithm and PPI network.
