# Supplementary material for: Epigenome-Wide Tobacco-Related Methylation Signature Identification and Their Multilevel Regulatory Network Inference for Lung Adenocarcinoma
Source: Biomed Res Int. 2020 Apr 24;2020:2471915. doi: 10.1155/2020/2471915 (PMC7201762; doi:10.1155/2020/2471915)
Supplement: Supplementary Materials — Figure S1: flow chart for the identification and classification of methylation biomarkers associated with smoking in lung adenocarcinoma. Figure S2: volcano plot of 95 signature genes in never-smokers versus smokers. Table S1: list of 105 signature methylation probes and their P values. Table S2: significantly enriched pathways of genes corresponding to 105 differential probes. Table S3: GO enrichment analysis of genes corresponding to 105 differential probes. Table S4: the relationship between methylation and expression. Table S5: the expression of 95 genes corresponding to 105 probes (current-smoker versus never-smoker). Table S6: related references and related types of 95 genes. Table S7: the 33 possible related genes corresponding to proteins whose betweenness is greater than or equal to 100 obtained by BIMC method using the shortest path algorithm and PPI network. [file 2471915.f1.pdf]

Epigenome-wide tobacco-related methylation signature identification and their multi-level regulatory network inference for lung adenocarcinoma

Supplementary Materials

Yan-Mei Dong[1], Ming Li[2], Qi-En He[1], Yi-Fan Tong[1], Ya-meng Wu, [2]Jun Hu[4], Ning Zhang[2*], Kai Song[1*]

1 School of Chemical Engineering and Technology, Tianjin University, 300072, Tianjin, China.

2 Department of Biomedical Engineering, Tianjin Key Lab of Biomedical Engineering Measurement, Tianjin University, 300072, Tianjin, China.

3 The Second Affiliated Hospital of Fujian Medical University, 362000, Quanzhou, Fujian, China.

4 Department of Hematology and Oncology, Karamay Central Hospital, 834000, Karamay, Xinjiang UygurAutonomous Region, China

*Corresponding authors:

Kai Song, School of Chemical Engineering and Technology, Tianjin University, Tianjin, 300072, China. Email: ksong@tju.edu.cn

Ning Zhang, Department of Biomedical Engineering, Tianjin Key Lab of Biomedical Engineering Measurement, Tianjin University, 300072 Tianjin, China

Email: zhni@tju.edu.cn

Partial least squares (Qiu, et al., 2017)

Partial least squares (PLS) is an efficient statistical regression technique that is highly suited for the analysis of high-dimensional data, a powerfully proven method for analyzing genomic and proteomic data, especially problems of classification and dimension reduction in bioinformatics and genomics(Abdi and Williams, 2013; Nguyen and Rocke, 2002; Song, et al., 2012).

Suppose that the data X is an $n \times p$ matrix of n samples and p genes (the raw data set should be scaled to zero mean and unit variance), and let **Y** denote the n×q vector of response values, such as the indicator of classification of smokers and non-smokers. When n<p, the usual regression tools such as ordinary least squares (OLS), cannot be applied since the p×p covariance matrix $\mathbf{X}^\mathbf{T}\mathbf{X}$ is singular. In contrast, PLS may be applied also to the cases, whose aims is to describe linear relationship between the predictor matrix $\mathbf{X} \in \mathbf{R}_{\mathbf{n}\times\mathbf{p}}$ and the response $\mathbf{Y} \in \mathbf{R}_{n\times q}$,

$$\mathbf{Y=XB+V} \tag{1}$$

where $\mathbf{B} \in \mathbf{R}_{\mathbf{p}\times\mathbf{q}}$ is the regression coefficient matrix and $\mathsf{V} \in \mathbf{R}_{\mathbf{n}\times\mathbf{q}}$ is the residual matrix. PLS regression is based on the basic principal component decomposition:

$$\mathbf{Y=TQ^T+F} \tag{2}$$

$$\mathbf{X=TP^T+E} \tag{3}$$

where $\mathbf{T} \in \mathbf{R}_{\mathbf{n}\times\mathbf{m}}$ is the latent variables (LVs) matrix, $\mathbf{P} \in \mathbf{R}_{\mathbf{p}\times\mathbf{m}}$ and $\mathbf{Q} \in \mathbf{R}_{\mathbf{q}\times\mathbf{m}}$ are matrices of coefficients, $\mathbf{E} \in \mathbf{R}_{\mathbf{n}\times\mathbf{p}}$ and $\mathbf{F} \in \mathbf{R}_{\mathbf{n}\times\mathbf{q}}$ are matrices of random errors, m is the number of LVs.

From equation (1), (2), and (3), the **T** is the key. The objective criterion for constructing components in PLS is to sequentially maximize the covariance between the response variable and a linear combination of the predictors. That is, in PLS, the components are constructed to maximize the objective criterion based on the sample covariance between **Y** and **XW**, thus,

$$w_k = \arg\max cov^2_{w^T w=1}(\boldsymbol{XW}, \boldsymbol{Y}) \tag{4}$$

Subject to the orthogonal constraint,

$$w_k^T \boldsymbol{X^T X W_i} = 0 \quad \text{for all} \quad 1 \le \mathrm{k} < \mathrm{i} \tag{5}$$

Where $\mathbf{W} \in \mathbf{R}_{\mathrm{p}\times\mathrm{m}}$is a matrix of weights.

To derive the T, PLS can all be seen as methods to construct a matrix of latent components T as a linear transformation of **X**,

$$\mathbf{T = XW} \tag{6}$$

If **T** is constructed, $\mathbf{Q^T}$ and is obtained as the least squares solution of Equation (2):

$$\mathbf{Q^T} = (\mathbf{T^T T})^{-1}\mathbf{T^T Y} \tag{7}$$

The matrix B regression coefficients matrix is constructed from Equation (1):

$$\mathbf{B} = \mathbf{W}(\mathbf{T^T T})^{-1}\mathbf{T^T Y} \tag{8}$$

The number of LVs is the only parameter of PLS which need to be decided, with the increase of LVs, the information of original data preserved is increasing, until reaching the maximal value, which is the rank of **X**, all the information of original data is contained in LVs.

Biweight midcorrelation coefficient algorithm (Yuan, et al., 2018)

Biweight midcorrelation is considered to be a good alternative to Pearson correlation coefficient since it is more robust to outliers.

In order to introduce the biweight midcorrelation coefficient (BIMC) of two numeric vectors $\mathbf{x} =$

$(x_1,\cdots,x_n)$ and $\mathbf{y}=(y_1,\cdots,y_n)$, **x** and **y** can be two column vectors of DNA methylation matrix, u_i, v_i are defined with i=1,…,n as follows:

$$u_i=\frac{x_i-med(\mathbf{x})}{T\cdot mad(\mathbf{x})} \tag{9}$$

$$v_i=\frac{y_i-med(\mathbf{y})}{T\cdot mad(\mathbf{y})} \tag{10}$$

$$mad(\mathbf{x})=med(|x_i-med(\mathbf{x})|) \tag{11}$$

where $med(\mathbf{x})$ and $med(\mathbf{y})$ are the median of vector **x** and **y** respectively. $mad(\cdot)$ represents the median absolute deviation of numeric vector. Based on u_i and v_i . The weights $w_i^{(x)}$ for x_i and $w_i^{(y)}$ for y_i are defined as follows:

$$w_i^{(x)}=\left(1-u_i^2\right)^2\mathbf{I}(1-|u_i|) \tag{12}$$

$$w_i^{(y)}=\left(1-v_i^2\right)^2\mathbf{I}(1-|v_i|) \tag{13}$$

where **I** is an indicator equation, for equation (13), the indicator equation $\mathbf{I}(1-|v_i|)$ is 1 if $(1-|v_i|)$>0 and otherwise equals to 0. The same situation occurs for equation (12). For equation (10) and (13), as the difference between y_i and $med(\mathbf{y})$ gets smaller and smaller, $w_i^{(y)}$ gets closer to 1. If the difference between y_i and $med(\mathbf{y})$ is larger than $\mathrm{T}\cdot mad(\mathbf{y})$, $w_i^{(y)}$ equals to 0. The same situation occurs for equation (9) and equation (12). T is a pre-defined parameter. Let us discuss pre-defined parameter T . In practice, the bigger T , the smaller the number of values to be filtered out. For T , we chose the highest valid value to include all potentially interesting values. In addition, users can determine T based on the data characteristics and possible proportion of outliers. The weight values of all outliers are guaranteed to be 0. Based on $w_i^{(x)}$ and $w_i^{(y)}$, we can define BIMC of vector **x** and **y** as follows:

$$pre(\mathbf{x},\mathbf{y})=\left(\frac{\sqrt{\sum_{i=1}^{n}[(x_i-med(\mathbf{x}))\cdot w_i^{(x)}]^2}}{\cdot\sqrt{\sum_{i=1}^{n}[(y_i-med(\mathbf{y}))\cdot w_i^{(y)}]^2}}\right)^{-1} \tag{14}$$

$$\mathrm{BIMC}(\mathbf{x},\mathbf{y})=\left(\sum_{i=1}^{n}(x_i-med(\mathbf{x}))\cdot w_i^{(x)}\cdot(y_i-med(\mathbf{y}))\cdot w_i^{(y)}\right)\cdot pre(\mathbf{x},\mathbf{y}) \tag{15}$$

where BIMC(**x, y**) represents the BIMC of **x** and ... It should be noted that, the range of BIMC is from -1 to 1. If there is a strong positive linear relationship between DNA methylation vectors, the value of BIMC will be close to 1. If there is a strong negative linear relationship between methylation vectors, the value of BIMC will be close to -1. If there is no linear relationship or only a weak linear relationship between methylation vectors, the value of BIMC will be 0 or close to 0.

Tobacco exposure Prediction Model assessment

SN (Sensitivity), SP (Specificity), ACC (Accuracy) are defined as follows:

$$\mathrm{SN} = \frac{TP}{TP + FN} \tag{16}$$

$$\mathrm{SP} = \frac{TN}{TN + FP} \tag{17}$$

$$\mathrm{ACC} = \frac{TP + TN}{TP + TN + FP + FN} \tag{18}$$

where TP, FP, TN and FN denote true positive, false positive, true negative, and false negative, respectively. In our study, smoker samples and non-smoker samples were designated as the positive and negative samples, respectively. Correspondingly, sensitivity is the proportion of smoker samples correctly classified, specificity is the proportion of non-smoker samples correctly classified, and accuracy is the proportion of both types of samples correctly classified. In this study, current-smokers were used as positive sample, and never-smokers were used as negative sample.

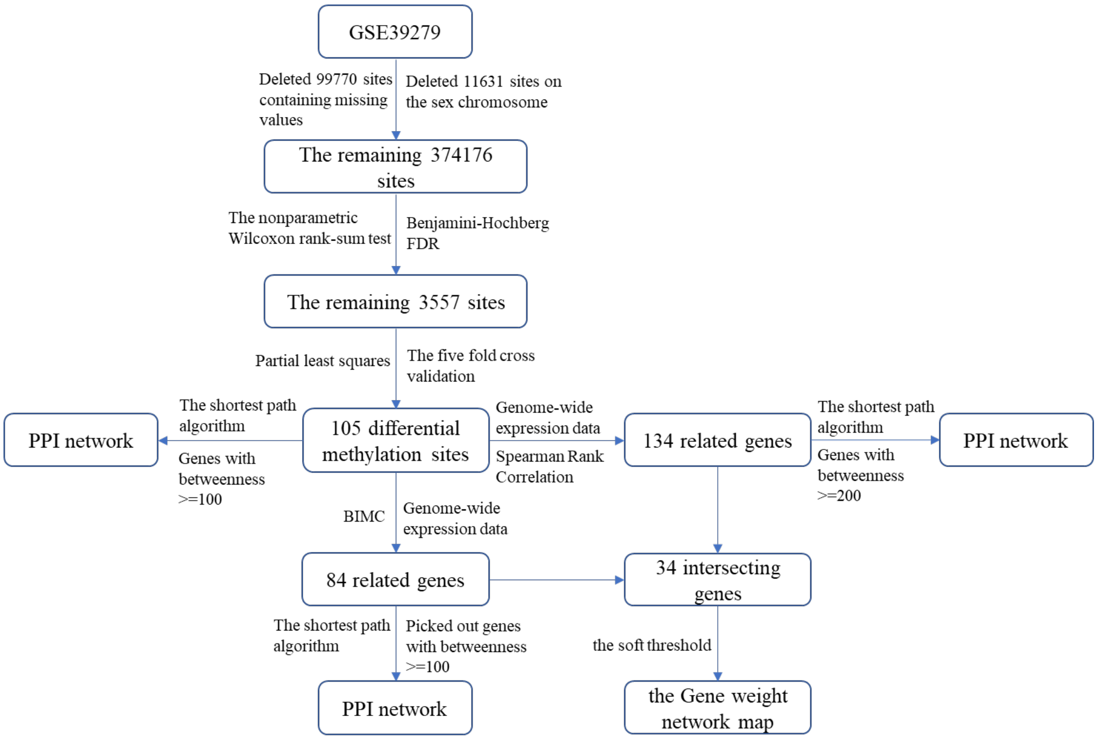

Fig. S1 Flow chart for identification and classification of methylation biomarkers associated with smoking in lung adenocarcinoma

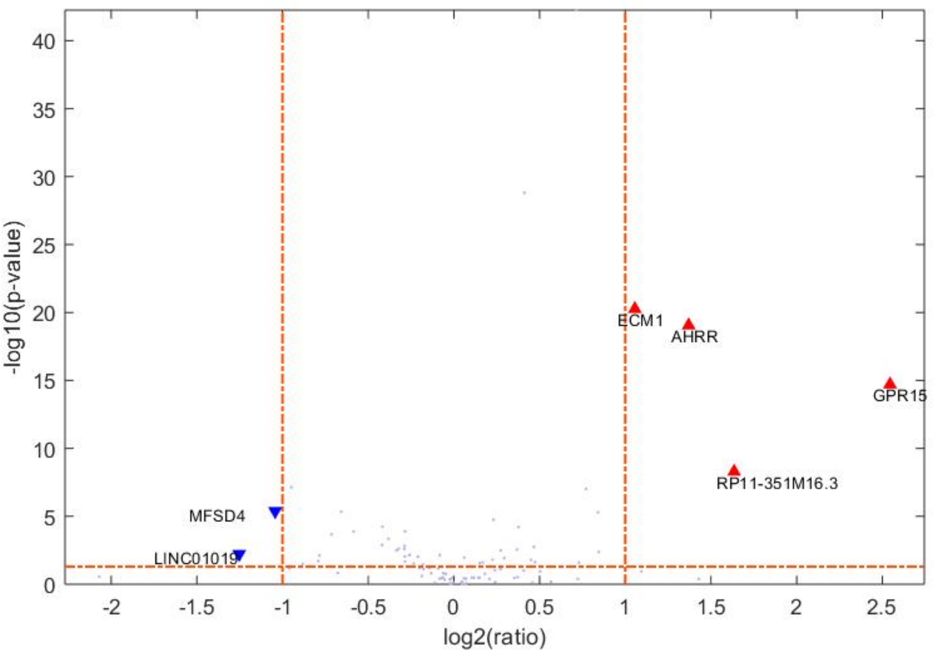

Fig. S2 Volcano plot of 95 signature genes in never-smokers vs smokers

Table S1. List of 105 signature methylation probes p-value

	Probe	Start	End	Gene_Symbol	CGI_Coordinate	Region	Feature_Type	Median value of smokers	Median value of non-smokers	p-value*
1	cg00334821	74094664	74094665	LIMK1	CGI:chr7:74083514-74084367	Body	.	0.21	0.16	4.22E-04
2	cg00370022	74722851	74722852	CYP1A1	CGI:chr15:74725845-74726995	Body	N_Shelf	0.62	0.69	9.02E-06
3	cg00688979	31145240	31145241	CCHCR1	CGI:chr6:31158308-31158784	Body	.	0.82	0.78	1.80E-05
4	cg00702638	44761801	44761802	KIAA1143;KIF15	CGI:chr3:44761360-44762126	TSS200	Island	0.04	0.06	1.70E-05
5	cg00976097	421618	421619	AHRR	CGI:chr5:421109-421636	Body	Island	0.93	0.90	1.31E-04
6	cg00993400	248859480	248859481	AL672294.1;ZNF692	CGI:chr1:248858212-248859220	TSS1500	S_Shore	0.14	0.17	2.60E-05
7	cg01049916	121517540	121517541	FGFR2	CGI:chr10:121597102-121598771	Body	.	0.80	0.83	3.82E-04
8	cg01637537	40021281	40021282	FAM83F	CGI:chr22:40021349-40021866	Body	N_Shore	0.64	0.70	1.85E-04
9	cg01971034	50330970	50330971	.	CGI:chr1:50332995-50333864	.	N_Shelf	0.68	0.74	3.14E-04
10	cg02050426	61554225	61554226	CDH20	CGI:chr18:61554218-61554753	Body	Island	0.74	0.79	1.29E-04
11	cg02387679	76404040	76404041	IQGAP2	CGI:chr5:76402934-76404335	Body	Island	0.19	0.14	2.81E-05
12	cg02498206	113268168	113268169	BOC	CGI:chr3:113211590-113212659	Body	.	0.80	0.84	9.35E-05
13	cg02826525	38959744	38959745	ARHGEF33;RP11-173C1.1	CGI:chr2:38959636-38960827	Body	Island	0.83	0.86	4.34E-05
14	cg02988118	42097957	42097958	.	CGI:chr2:42101349-42101687	.	N_Shelf	0.86	0.84	8.70E-05
15	cg03078488	23467029	23467030	IGF2BP3	CGI:chr7:23468565-23470093	Body	N_Shore	0.80	0.83	2.84E-04
16	cg03277049	156816287	156816288	LINC00886	CGI:chr3:156816050-156817342	.	Island	0.24	0.34	3.46E-05
17	cg03642695	15308641	15308642	TEKT3	CGI:chr17:15341388-15341809	Body	.	0.81	0.86	1.08E-04
18	cg03789372	27268239	27268240	.	CGI:chr8:27287614-27288084	.	.	0.61	0.67	1.79E-04
19	cg03806812	85953713	85953714	.	CGI:chr2:85888467-85889204	.	.	0.52	0.45	2.44E-04
20	cg03945895	13784857	13784858	PRDM2	CGI:chr1:13748772-13749963	Body	.	0.88	0.86	3.60E-05
21	cg03985801	202213475	202213476	LGR6	CGI:chr1:202214065-202214535	TSS1500	N_Shore	0.47	0.54	4.85E-05
22	cg04267214	38804378	38804379	.	CGI:chr1:38804057-38804579	.	Island	0.17	0.13	3.95E-04
23	cg04616529	11088129	11088130	CLEC16A	CGI:chr16:11174035-11174354	Body	.	0.83	0.79	1.92E-04
24	cg04865290	52893532	52893533	TMEM110;TMEM110-MUSTN1	CGI:chr3:52897210-52897824	Body	N_Shelf	0.71	0.63	4.10E-05
25	cg05033369	161706679	161706680	FCRLA	CGI:chr1:161725847-161727508	TSS1500	.	0.29	0.41	9.98E-06
26	cg05559381	123874771	123874772	.	CGI:chr5:123636064-123636314	.	.	0.73	0.66	1.99E-04
27	cg05575921	373263	373264	AHRR	CGI:chr5:373727-374311	Body	N_Shore	0.61	0.68	4.43E-04
28	cg05752786	202710627	202710628	SYT2	CGI:chr1:202709795-202710641	TSS1500	Island	0.15	0.08	1.85E-04
29	cg05787209	31011200	31011201	STX1B	CGI:chr16:31010457-31011052	TSS1500	S_Shore	0.58	0.64	3.70E-04
30	cg05951221	232419692	232419693	ECEL1P1	CGI:chr2:232418687-232421249	.	Island	0.33	0.40	7.95E-05
31	cg06010163	156564851	156564852	.	CGI:chr6:156631022-156631312	.	.	0.36	0.31	1.06E-04
32	cg06227763	10447087	10447088	.	CGI:chr18:10454085-10455271	.	.	0.74	0.79	3.52E-04
33	cg06540950	124574637	124574638	.	CGI:chr5:124651705-124652055	.	.	0.80	0.70	1.16E-04
34	cg06637330	3587737	3587738	.	CGI:chr5:3590530-3591886	.	N_Shelf	0.34	0.46	1.06E-04

35	cg07160783	64827508	64827509	.	CGI:chr16:64950778-64951005	.	.	0.69	0.78	3.19E-04
36	cg07325233	33266357	33266358	AP000295.9;IL10RB;IL10RB-AS1	CGI:chr21:33266210-33266770	TSS200	Island	0.04	0.04	2.36E-04
37	cg07709148	29529721	29529722	RP11-486M23.1	CGI:chr8:29352966-29353284	.	.	0.14	0.10	5.22E-05
38	cg07813142	170716713	170716714	SP5	CGI:chr2:170713367-170717394	Body	Island	0.37	0.56	1.24E-04
39	cg08008475	27403642	27403643	RNY1P1	CGI:chr13:27424449-27425041	.	.	0.76	0.69	8.86E-05
40	cg08374798	62815701	62815702	COL9A3	CGI:chr20:62816333-62817666	TSS1500	N_Shore	0.55	0.47	1.00E-04
41	cg08733957	23798808	23798809	GALE	CGI:chr1:23800300-23801059	Body	N_Shore	0.77	0.70	2.24E-04
42	cg08894131	147758953	147758954	GJA5	CGI:chr1:147669720-147670796	Body	.	0.73	0.80	4.73E-04
43	cg09194449	157690222	157690223	PTPRN2	CGI:chr7:157688558-157694027	Body	Island	0.30	0.23	7.00E-05
44	cg09278187	42178361	42178362	FOXJ3	CGI:chr1:42145814-42146020	3'UTR	.	0.71	0.60	1.59E-04
45	cg09370982	2497558	2497559	RP11-20I23.1;TBC1D24	CGI:chr16:2496263-2497038	Body	S_Shore	0.71	0.62	1.67E-04
46	cg09799983	38074613	38074614	CYP1B1;CYP1B1-AS1	CGI:chr2:38074133-38077376	Body	Island	0.28	0.54	6.04E-08
47	cg10076730	110438086	110438087	COL4A2	CGI:chr13:110438290-110438513	Body	N_Shore	0.75	0.78	4.76E-05
48	cg10354195	132375285	132375286	LRRC27	CGI:chr10:132388102-132388909	3'UTR	.	0.83	0.78	3.82E-04
49	cg10385390	7962445	7962446	PARK7	CGI:chr1:7961241-7962166	5'UTR	S_Shore	0.29	0.34	2.79E-04
50	cg10413224	34079146	34079147	BMPER	CGI:chr7:34078851-34079147	Body	Island	0.68	0.74	1.24E-05
51	cg10650290	158386935	158386936	PTPRN2	CGI:chr7:158405853-158406215	Body	.	0.61	0.70	2.56E-04
52	cg11545521	47997915	47997916	PTPRJ	CGI:chr11:47980052-47981618	Body	.	0.68	0.57	4.53E-07
53	cg11751707	38075444	38075445	CYP1B1;CYP1B1-AS1	CGI:chr2:38074133-38077376	5'UTR	Island	0.12	0.26	1.20E-08
54	cg11954332	170670148	170670149	PRRX1	CGI:chr1:170664161-170665037	Body	.	0.47	0.54	3.35E-04
55	cg12020590	134656427	134656428	.	CGI:chr11:134664348-134664563	.	.	0.65	0.76	3.53E-05
56	cg12387247	7702088	7702089	FCER2	CGI:chr19:7680604-7682828	1stExon	.	0.52	0.60	8.55E-05
57	cg13563863	3506441	3506442	FZR1	CGI:chr19:3505822-3506772	5'UTR	Island	0.08	0.09	4.36E-04
58	cg13654445	85021468	85021469	NTRK2	CGI:chr9:84668263-84670789	3'UTR	.	0.84	0.87	8.86E-05
59	cg13990746	173669905	173669906	ANKRD45	CGI:chr1:173669523-173669906	TSS200	Island	0.29	0.17	2.52E-04
60	cg14270346	38026079	38026080	RP11-613M10.9;SHB	CGI:chr9:38067686-38069631	Body	.	0.54	0.44	2.88E-04
61	cg14320852	87265792	87265793	.	CGI:chr9:87148421-87148852	.	.	0.74	0.80	1.51E-04
62	cg14373988	2413894	2413895	PEX10	CGI:chr1:2414452-2414717	TSS1500	N_Shore	0.67	0.79	1.43E-04
63	cg14419740	157690643	157690644	PTPRN2	CGI:chr7:157688558-157694027	Body	Island	0.40	0.23	4.15E-04
64	cg15233380	26046474	26046475	SHISA2	CGI:chr13:26050587-26052127	3'UTR	.	0.75	0.80	2.56E-04
65	cg15513657	100778269	100778270	MEG3	CGI:chr14:100734280-100734592	.	.	0.79	0.83	3.82E-04
66	cg15585555	206559387	206559388	RASSF5	CGI:chr1:206557069-206557580	Body	S_Shore	0.49	0.37	3.46E-05
67	cg15680620	173669991	173669992	ANKRD45	CGI:chr1:173669523-173669906	TSS200	S_Shore	0.43	0.33	2.61E-04
68	cg15922705	70281453	70281454	COL9A1	CGI:chr6:70282337-70283209	Body	N_Shore	0.74	0.82	5.22E-05
69	cg16315376	202710634	202710635	SYT2	CGI:chr1:202709795-202710641	TSS1500	Island	0.25	0.17	1.63E-04
70	cg16322479	444113	444114	EXOC3;EXOC3-AS1	CGI:chr5:442705-443489	5'UTR	S_Shore	0.62	0.72	2.44E-04

71	cg16377959	3511267	3511268	LINC01019	CGI:chr5:3535516-3536241	.	.	0.82	0.87	9.87E-05
72	cg16840978	37896037	37896038	RAB11FIP1	CGI:chr8:37898936-37899821	Body	N_Shelf	0.74	0.65	3.44E-06
73	cg16884847	45894349	45894350	PRKCE	CGI:chr2:45650039-45652546	Body	.	0.73	0.58	1.73E-04
74	cg17211612	123846100	123846101	DNAH10	CGI:chr12:123787581-123788038	Body	.	0.80	0.92	9.87E-05
75	cg17373442	143121149	143121150	CHST2	CGI:chr3:143119043-143121996	Body	Island	0.33	0.12	3.19E-04
76	cg17676618	78803664	78803665	.	CGI:chr10:78973556-78974256	.	.	0.55	0.64	4.29E-04
77	cg18001059	42932481	42932482	MRPL32;PSMA2	CGI:chr7:42932035-42932700	1stExon	Island	0.05	0.06	6.88E-05
78	cg18713316	154760384	154760385	KCNN3	CGI:chr1:154760346-154760960	Body	Island	0.38	0.31	3.41E-04
79	cg18883807	96027662	96027663	.	CGI:chr14:96038974-96040065	.	.	0.17	0.22	4.15E-04
80	cg18919659	42947078	42947079	PACSIN2	CGI:chr22:43014415-43015504	TSS200	.	0.62	0.54	1.79E-04
81	cg19341901	149883790	149883791	.	CGI:chr7:149873277-149874300	.	.	0.65	0.54	2.93E-04
82	cg19859270	98532450	98532451	CPOX;GPR15	CGI:chr3:98522565-98523091	1stExon	.	0.79	0.83	1.20E-06
83	cg20439473	57983709	57983710	VEZF1	CGI:chr17:57987469-57988690	Body	N_Shelf	0.83	0.76	3.95E-04
84	cg20459495	158923446	158923447	.	CGI:chr2:158968342-158969889	.	.	0.50	0.42	1.77E-05
85	cg20538211	57161121	57161122	IGFBP7-AS1	CGI:chr4:57163675-57164360	.	N_Shelf	0.51	0.58	1.51E-04
86	cg20546279	152894924	152894925	.	CGI:chr7:152894373-152894621	.	S_Shore	0.59	0.66	3.52E-04
87	cg20628376	28469158	28469159	RP11-351M16.3	CGI:chr10:28532260-28534163	.	.	0.43	0.51	4.50E-05
88	cg21012061	205599429	205599430	ELK4;MFSD4	CGI:chr1:205591961-205592345	Body	.	0.64	0.51	2.07E-05
89	cg21083936	2273860	2273861	.	CGI:chr11:2268874-2271702	.	S_Shelf	0.68	0.58	3.76E-04
90	cg21500300	24949587	24949588	BCAT1;RP11-662I13.2	CGI:chr12:24948673-24949139	TSS1500	S_Shore	0.64	0.72	1.79E-04
91	cg21885107	73245581	73245582	PAPLN;RP4-647C14.2	CGI:chr14:73245580-73246203	Body	Island	0.45	0.51	2.40E-04
92	cg23369748	148384526	148384527	SASH1	CGI:chr6:148342289-148343079	Body	.	0.77	0.80	3.82E-04
93	cg23501962	35418705	35418706	RP4-683L5.1;SLC1A2	CGI:chr11:35418874-35420335	Body	N_Shore	0.21	0.17	4.15E-04
94	cg23854567	120257090	120257091	PXN	CGI:chr12:120265173-120265738	Body	.	0.59	0.47	4.50E-05
95	cg24203542	19440737	19440738	NAV2	CGI:chr11:19346350-19346687	Body	.	0.77	0.67	2.61E-04
96	cg24279017	11724806	11724807	ETV6	CGI:chr12:11648919-11649619	Body	.	0.59	0.50	8.86E-05
97	cg24772753	170716909	170716910	SP5	CGI:chr2:170713367-170717394	Body	Island	0.46	0.57	1.20E-04
98	cg25192619	37499628	37499629	CCDC167	CGI:chr6:37499619-37500024	Body	Island	0.16	0.18	3.82E-04
99	cg26005485	138496253	138496254	FAM135B	CGI:chr8:138496552-138497531	5'UTR	N_Shore	0.50	0.56	1.89E-04
100	cg26029292	144830196	144830197	ZNF7	CGI:chr8:144826448-144827907	Body	S_Shelf	0.70	0.77	3.70E-04
101	cg26076054	421202	421203	AHRR	CGI:chr5:421109-421636	Body	Island	0.88	0.80	2.48E-04
102	cg26582784	92836028	92836029	AC002454.1;CDK6	CGI:chr7:92832887-92837073	5'UTR	Island	0.09	0.08	2.20E-04
103	cg26799398	150507033	150507034	ECM1;TARS2	CGI:chr1:150515560-150515940	TSS1500	.	0.83	0.77	2.88E-04
104	cg26972614	71999839	71999840	IL18BP	CGI:chr11:72080339-72080914	5'UTR	.	0.48	0.40	9.02E-06
105	cg27052537	134638283	134638284	.	CGI:chr7:134458363-134459311	.	.	0.61	0.48	1.08E-04

* Wilcoxon rank-sum test

Table S2. Significantly enriched pathways of genes corresponding to 105 differential probes

<table>
<tr><th>#Term</th><th>ID</th><th>P-Value</th><th>Corrected P-Value</th><th>Input</th></tr>
<tr><td>ECM-receptor interaction</td><td>hsa04512</td><td>0.000761</td><td>0.012246044</td><td>1284|1299|1297</td></tr>
<tr><td>PI3K-Akt signaling pathway</td><td>hsa04151</td><td>0.000811</td><td>0.012741464</td><td>2263|1299|1021|1297|1284</td></tr>
<tr><td>Focal adhesion</td><td>hsa04510</td><td>0.000944</td><td>0.014020672</td><td>5829|1284|1299|1297</td></tr>
<tr><td>Protein digestion and absorption</td><td>hsa04974</td><td>0.000988</td><td>0.01435258</td><td>1284|1299|1297</td></tr>
<tr><td>Regulation of actin cytoskeleton</td><td>hsa04810</td><td>0.001162</td><td>0.015962134</td><td>5829|2263|10788|3984</td></tr>
<tr><td>Tryptophan metabolism</td><td>hsa00380</td><td>0.003497</td><td>0.031923526</td><td>1545|1543</td></tr>
<tr><td>Ovarian steroidogenesis</td><td>hsa04913</td><td>0.005314</td><td>0.040827449</td><td>1545|1543</td></tr>
<tr><td>Non-small cell lung cancer</td><td>hsa05223</td><td>0.006571</td><td>0.046783375</td><td>83593|1021</td></tr>
<tr><td>Steroid hormone biosynthesis</td><td>hsa00140</td><td>0.007017</td><td>0.048651705</td><td>1545|1543</td></tr>
</table>

Table S3. GO enrichment analysis of genes corresponding to 105 differential probes

<table>
<tr><th></th><th>#Term</th><th>ID</th><th>P-Value</th><th>Corrected P-Value</th><th>Gene ID</th></tr>
<tr><td>1</td><td>single-organism process</td><td>GO:0044699</td><td>3.15E-16</td><td>9.18E-13</td><td>64518|51059|5396|57465|127833|83593|10068|2838|23328|7799|11336|6506|196385|1893|91653|80223|80222|1543|7716|5829|51343|89797|5192|1371|387914|1299|7553|2263|100526772|1297|5683|586|1545|2582|2120|3588|10643|2702|84824|59352|100271715|11315|10788|4915|3782|1284|1021|112755|6461|375346|5581|11252|64983|2208|5795|389058|56992|2005|3984|9435|168667|54535|5799</td></tr>
<tr><td>2</td><td>cellular process</td><td>GO:0009987</td><td>1.56E-14</td><td>2.28E-11</td><td>64518|51059|5396|57465|127833|83593|10068|2838|23328|7799|11336|6506|5799|196385|1893|91653|80223|80222|1543|7716|5829|51343|5192|1371|1299|7553|2263|100526772|1297|5683|89932|586|1545|2120|3588|10643|2702|23274|84824|59352|100271715|11315|10788|57491|4915|3782|1284|1021|112755|6461|375346|5581|11252|64983|2208|5795|389058|56992|55657|2005|3984|9435|168667|54535|22887</td></tr>
<tr><td>3</td><td>single-organism cellular process</td><td>GO:0044763</td><td>2.18E-12</td><td>2.12E-09</td><td>64518|51059|5396|57465|127833|83593|10068|2838|23328|11336|6506|196385|1893|91653|80223|80222|1543|7716|5829|51343|5192|1371|1299|2263|100526772|1297|5683|586|1545|2120|3588|2702|84824|59352|100271715|11315|10788|4915|3782|1284|1021|112755|6461|375346|5581|11252|64983|2208|5795|56992|2005|3984|168667|54535|5799</td></tr>
<tr><td>4</td><td>binding</td><td>GO:0005488</td><td>5.35E-12</td><td>3.89E-09</td><td>64518|4915|5396|57465|127833|83593|10068|23328|7799|6506|196385|1893|91653|80223|80222|1543|7716|5829|51343|89797|5192|137</td></tr>
</table>

<table>
<tr><td></td><td></td><td></td><td></td><td></td><td>1|7553|2263|1297|5683|89932|57456|1545|2582|339416|2120|3588|10643|2702|57491|59352|11336|11315|10788|586|3782|1284|1021|112755|6461|375346|5581|11252|64983|28316|2208|5795|389058|56992|55657|2005|3984|54535|22887</td></tr>
<tr><td>5</td><td>cell part</td><td>GO:0044464</td><td>2.24E-11</td><td>1.18E-08</td><td>64518|4915|5396|57465|127833|83593|56992|11336|6506|7799|5799|196385|1893|91653|80223|80222|1543|7716|5829|51343|89797|5192|1371|387914|1299|7553|2263|100526772|1297|5683|586|1545|2582|2120|3588|10643|2702|23274|84824|59352|2838|11315|10788|57491|3782|1284|1021|112755|6461|375346|5581|11252|64983|28316|2208|5795|389058|55657|2005|3984|9435|54535|22887</td></tr>
<tr><td>6</td><td>cell</td><td>GO:0005623</td><td>2.43E-11</td><td>1.18E-08</td><td>64518|4915|5396|57465|127833|83593|56992|11336|6506|7799|5799|196385|1893|91653|80223|80222|1543|7716|5829|51343|89797|5192|1371|387914|1299|7553|2263|100526772|1297|5683|586|1545|2582|2120|3588|10643|2702|23274|84824|59352|2838|11315|10788|57491|3782|1284|1021|112755|6461|375346|5581|11252|64983|28316|2208|5795|389058|55657|2005|3984|9435|54535|22887</td></tr>
<tr><td>7</td><td>developmental process</td><td>GO:0032502</td><td>1.59E-10</td><td>6.59E-08</td><td>64518|5396|57465|127833|23328|7799|1893|91653|1545|1543|7716|51343|89797|387914|1299|7553|2263|100526772|1297|5683|2120|10643|2702|84824|1284|1021|6461|4915|11252|6506|389058|2005|3984|9435|168667|54535</td></tr>
<tr><td>8</td><td>single-organism developmental process</td><td>GO:0044767</td><td>4.67E-10</td><td>1.62E-07</td><td>64518|5396|57465|127833|23328|7799|1893|91653|1545|1543|7716|51343|89797|387914|1299|7553|2263|100526772|1297|5683|2120|2702|84824|1284|1021|6461|4915|11252|6506|389058|2005|3984|9435|168667|54535</td></tr>
<tr><td>9</td><td>anatomical structure development</td><td>GO:0048856</td><td>5.01E-10</td><td>1.62E-07</td><td>64518|5396|57465|127833|23328|7799|1893|91653|1545|1543|7716|51343|89797|387914|1299|7553|2263|100526772|1297|5683|2120|10643|2702|389058|1284|1021|6461|4915|11252|6506|3984|9435|168667|54535</td></tr>
<tr><td>10</td><td>biological regulation</td><td>GO:0065007</td><td>8.60E-10</td><td>2.50E-07</td><td>64518|4915|5396|57465|127833|83593|10068|100271715|23328|7799|5799|1893|91653|80223|57491|1543|7716|5829|51343|89797|89932|7553|2263|5683|1545|2120|3588|10643|2702|389058|59352|2838|11315|10788|3782|1284|1021|112755|6461|375346|5581|11252|6506|2208|5795|55657|2005|3984|168667|22887</td></tr>
<tr><td>11</td><td>single-multicellular organism process</td><td>GO:0044707</td><td>1.32E-09</td><td>3.48E-07</td><td>4915|5396|57465|127833|23328|7799|1893|91653|80223|1545|1543|7716|51343|89797|387914|1299|7553|2263|100526772|1297|5683|2120|10643|2702|389058|11315|1284|1021|6461|5581|6506|3984|9435|168667|54535</td></tr>
<tr><td>12</td><td>regulation of biological process</td><td>GO:0050789</td><td>1.70E-09</td><td>4.12E-07</td><td>64518|4915|5396|127833|83593|10068|100271715|23328|7799|5799|1893|91653|80223|57491|1543|7716|5829|51343|89932|7553|2263|5683|1545|2120|3588|10643|2702|389058|59352|2838|11315|10788|3782|1284|1021|112755|6461|375346|5581|11252|6506|2208|5795|55657|2005|3984|168667|22887</td></tr>
<tr><td>13</td><td>membrane-bounded organelle</td><td>GO:0043227</td><td>2.20E-09</td><td>4.92E-07</td><td>64518|4915|5396|127833|83593|10068|7799|5799|1893|80223|80222|1543|7716|5829|51343|89797|5192|1371|387914|1299|7553|2263|100526772|1297|5683|1545|2582|2120|3588|10643|112755|23274|389058|59352|11336|11315|10788|1284|1021|375346|5581|11252|64983|2208|5795|55657|2005|3984|9435|54535|22887</td></tr>
<tr><td>14</td><td>organelle</td><td>GO:0043226</td><td>2.48E-09</td><td>5.17E-07</td><td>64518|4915|5396|127833|83593|10068|7799|5799|196385|1893|80223|80222|1543|7716|5829|51343|89797|5192|1371|387914|1299|7553</td></tr>
</table>

<table>
<tr><td></td><td></td><td></td><td></td><td></td><td>|2263|100526772|1297|5683|1545|2582|2120|3588|10643|112755|23274|389058|59352|11336|11315|10788|1284|1021|375346|5581|11252|64983|2208|5795|56992|55657|2005|3984|9435|54535|22887</td></tr>
<tr><td>15</td><td>multicellular organism development</td><td>GO:0007275</td><td>2.96E-09</td><td>5.74E-07</td><td>5396|57465|127833|23328|7799|1893|91653|1545|1543|7716|51343|89797|387914|1299|7553|2263|100526772|1297|5683|2120|2702|389058|1284|1021|6461|4915|6506|3984|9435|168667|54535</td></tr>
<tr><td>16</td><td>multicellular organismal process</td><td>GO:0032501</td><td>5.04E-09</td><td>9.18E-07</td><td>4915|5396|57465|127833|23328|7799|1893|91653|80223|1545|1543|7716|5829|51343|89797|387914|1299|7553|2263|100526772|1297|5683|2120|10643|2702|389058|11315|1284|1021|6461|5581|6506|5795|3984|9435|168667|54535</td></tr>
<tr><td>17</td><td>intracellular part</td><td>GO:0044424</td><td>6.15E-09</td><td>1.05E-06</td><td>64518|4915|5396|57465|127833|83593|56992|7799|5799|196385|1893|80223|80222|1543|7716|5829|51343|89797|5192|1371|387914|1299|7553|2263|100526772|1297|5683|586|1545|2582|2120|10643|112755|23274|84824|59352|11336|11315|10788|57491|1284|1021|6461|375346|5581|11252|64983|389058|55657|2005|3984|9435|54535|22887</td></tr>
<tr><td>18</td><td>blood vessel morphogenesis</td><td>GO:0048514</td><td>7.49E-09</td><td>1.21E-06</td><td>1284|1545|5396|7716|6461|4915|23328|2263|2702|168667|1893</td></tr>
<tr><td>19</td><td>protein binding</td><td>GO:0005515</td><td>9.20E-09</td><td>1.39E-06</td><td>64518|5396|57465|127833|83593|10068|23328|1893|91653|80223|57491|1543|5581|5829|51343|89797|5192|1371|2263|56992|5683|57456|2582|339416|2120|3588|10643|2702|59352|11336|11315|10788|586|3782|1284|1021|112755|6461|375346|4915|11252|6506|2208|5795|2005|3984|54535</td></tr>
<tr><td>20</td><td>intracellular organelle part</td><td>GO:0044446</td><td>9.58E-09</td><td>1.39E-06</td><td>64518|5396|127833|83593|56992|196385|1893|80223|80222|1543|7716|5829|51343|89797|5192|1371|387914|1299|2263|1297|5683|2120|112755|23274|1545|59352|11336|11315|10788|1284|1021|375346|4915|11252|64983|2005|3984|9435|54535|5799</td></tr>
<tr><td>21</td><td>intracellular</td><td>GO:0005622</td><td>1.68E-08</td><td>2.33E-06</td><td>64518|4915|5396|57465|127833|83593|56992|7799|5799|196385|1893|80223|80222|1543|7716|5829|51343|89797|5192|1371|387914|1299|7553|2263|100526772|1297|5683|586|1545|2582|2120|10643|112755|23274|84824|59352|11336|11315|10788|57491|1284|1021|6461|375346|5581|11252|64983|389058|55657|2005|3984|9435|54535|22887</td></tr>
<tr><td>22</td><td>organelle part</td><td>GO:0044422</td><td>1.84E-08</td><td>2.43E-06</td><td>64518|5396|127833|83593|56992|196385|1893|80223|80222|1543|7716|5829|51343|89797|5192|1371|387914|1299|2263|1297|5683|2120|112755|23274|1545|59352|11336|11315|10788|1284|1021|375346|4915|11252|64983|2005|3984|9435|54535|5799</td></tr>
<tr><td>23</td><td>blood vessel development</td><td>GO:0001568</td><td>3.80E-08</td><td>4.81E-06</td><td>1284|1545|5396|7716|6461|4915|23328|2263|2702|168667|1893</td></tr>
<tr><td>24</td><td>vasculature development</td><td>GO:0001944</td><td>5.34E-08</td><td>6.37E-06</td><td>1284|1545|5396|7716|6461|4915|23328|2263|2702|168667|1893</td></tr>
<tr><td>25</td><td>regulation of cellular process</td><td>GO:0050794</td><td>5.47E-08</td><td>6.37E-06</td><td>4915|5396|127833|83593|10068|100271715|23328|7799|1893|91653|80223|57491|1543|7716|5829|51343|89932|7553|2263|5683|1545|3588|10643|2702|389058|59352|2838|11315|10788|3782|2120|1021|112755|6461|375346|5581|11252|2208|5795|55657|2005|3984|168667|22887</td></tr>
<tr><td>26</td><td>intracellular organelle</td><td>GO:0043229</td><td>6.76E-08</td><td>7.49E-06</td><td>64518|4915|5396|127833|83593|56992|7799|5799|196385|1893|80223|80222|1543|7716|5829|51343|89797|5192|1371|387914|1299|7553</td></tr>
</table>

					|2263|100526772|1297|5683|1545|2120|10643|112755|23274|389058|59352|11336|11315|10788|1284|1021|375346|5581|11252|64983|55657|2005|3984|9435|54535|22887
27	cytoplasm	GO:0005737	6.95E-08	7.49E-06	64518|57465|127833|83593|56992|196385|1893|80223|80222|1543|5581|5829|51343|5192|1371|387914|1299|2263|1297|5683|586|1545|2582|2120|10643|112755|23274|84824|59352|11336|11315|10788|57491|1284|1021|6461|375346|4915|11252|64983|2005|3984|9435|54535|5799
28	cytoplasmic part	GO:0044444	9.19E-08	9.55E-06	64518|127833|56992|1893|80223|80222|1543|5581|5829|51343|5192|1371|387914|1299|2263|1297|5683|586|2582|10643|112755|23274|1545|59352|11336|11315|10788|1284|1021|6461|375346|4915|11252|64983|3984|9435|54535|5799
29	intracellular membrane-bounded organelle	GO:0043231	1.28E-07	1.29E-05	64518|4915|5396|127833|83593|7799|5799|1893|80223|80222|1543|7716|5829|51343|89797|5192|1371|387914|1299|7553|2263|100526772|1297|5683|1545|2120|10643|112755|23274|389058|59352|11336|11315|1284|1021|375346|5581|11252|64983|55657|2005|3984|9435|54535|22887
30	response to stimulus	GO:0050896	1.69E-07	1.64E-05	4915|5396|83593|10068|100271715|23328|7799|1893|91653|57491|1543|7716|5829|51343|1371|2263|100526772|5683|1545|3588|112755|389058|59352|2838|11315|10788|1284|1021|6461|375346|5581|6506|2208|5795|3984|9435|168667|5799
31	anatomical structure morphogenesis	GO:0009653	1.82E-07	1.71E-05	91653|1545|5396|7716|127833|6461|4915|11252|10643|2702|64518|1284|389058|3984|2263|23328|168667|1297|5683|1893
32	system development	GO:0048731	2.46E-07	2.24E-05	5396|57465|127833|23328|1893|91653|1545|1543|7716|51343|89797|1299|2263|100526772|1297|5683|2120|2702|389058|1284|1021|6461|4915|6506|3984|168667
33	angiogenesis	GO:0001525	2.64E-07	2.33E-05	1284|1545|7716|6461|23328|2263|2702|168667|1893
34	regulation of localization	GO:0032879	4.40E-07	3.77E-05	3782|80223|1545|1021|112755|5581|127833|83593|375346|4915|11252|6506|23328|5795|59352|11315|2702|168667|1893
35	endomembrane system	GO:0012505	5.37E-07	4.28E-05	64518|127833|1893|80223|1545|1543|5581|51343|387914|1299|2263|1297|112755|23274|59352|11336|11315|1284|375346|4915|11252|3984|9435|5799
36	single organism signaling	GO:0044700	5.44E-07	4.28E-05	5396|127833|83593|10068|100271715|23328|1893|91653|80223|1545|5581|5829|2263|5683|3588|2702|59352|2838|11315|10788|1021|112755|6461|375346|4915|6506|2208|5795|3984|168667|5799
37	signaling	GO:0023052	5.56E-07	4.28E-05	5396|127833|83593|10068|100271715|23328|1893|91653|80223|1545|5581|5829|2263|5683|3588|2702|59352|2838|11315|10788|1021|112755|6461|375346|4915|6506|2208|5795|3984|168667|5799
38	positive regulation of cell communication	GO:0010647	5.58E-07	4.28E-05	1545|5396|112755|5581|6461|4915|23328|5795|59352|2263|11315|2702|168667|5683|1893

<table>
<tr><td></td><td></td><td></td><td></td><td></td><td></td></tr>
<tr><td>39</td><td>cell communication</td><td>GO:0007154</td><td>6.03E-07</td><td>4.50E-05</td><td>5396|127833|83593|10068|100271715|23328|1893|91653|80223|1545|5581|5829|2263|5683|3588|2702|59352|2838|11315|10788|1021|112755|6461|375346|4915|6506|2208|5795|3984|168667|5799</td></tr>
<tr><td>40</td><td>positive regulation of biological process</td><td>GO:0048518</td><td>7.15E-07</td><td>5.21E-05</td><td>5396|127833|83593|23328|1893|91653|1545|1543|5581|2208|51343|2263|5683|2702|59352|11315|10788|3782|2120|1021|112755|6461|375346|4915|6506|5795|3984|168667</td></tr>
<tr><td>41</td><td>negative regulation of biological process</td><td>GO:0048519</td><td>8.83E-07</td><td>6.27E-05</td><td>5396|83593|10068|1893|80223|57491|5581|51343|89932|2263|5683|1284|10643|2702|1545|11315|10788|2120|1021|4915|11252|5795|2005|3984|168667|5799</td></tr>
<tr><td>42</td><td>positive regulation of signal transduction</td><td>GO:0009967</td><td>1.12E-06</td><td>7.74E-05</td><td>1545|5396|5581|6461|4915|23328|5795|59352|2263|11315|112755|168667|5683|1893</td></tr>
<tr><td>43</td><td>regulation of protein ubiquitination</td><td>GO:0031396</td><td>1.17E-06</td><td>7.93E-05</td><td>5581|83593|51343|23328|3984|11315|5683</td></tr>
<tr><td>44</td><td>cell surface receptor signaling pathway</td><td>GO:0007166</td><td>1.28E-06</td><td>8.46E-05</td><td>91653|1021|5396|5581|5829|6461|4915|23328|2208|5795|59352|3984|2263|11315|112755|3588|168667|5683|1893</td></tr>
<tr><td>45</td><td>localization</td><td>GO:0051179</td><td>1.49E-06</td><td>9.62E-05</td><td>64518|127833|83593|56992|2838|23328|1893|80223|1545|5581|5192|10643|2702|59352|11336|11315|112755|3782|1021|375346|4915|11252|6506|5795|3984|148808|168667|54535|5799</td></tr>
<tr><td>46</td><td>membrane</td><td>GO:0016020</td><td>1.62E-06</td><td>0.000103</td><td>64518|127833|56992|11336|6506|91653|80223|1545|1543|5581|5829|51343|5192|1371|387914|2263|100526772|28316|3588|2702|23274|59352|2838|11315|112755|3782|6461|375346|4915|11252|64983|2208|5795|3984|148808|9435|154467|5799</td></tr>
<tr><td>47</td><td>positive regulation of response to stimulus</td><td>GO:0048584</td><td>1.90E-06</td><td>0.000117</td><td>1545|5396|5581|2208|6461|4915|23328|5795|59352|3984|2263|11315|112755|168667|5683|1893</td></tr>
<tr><td>48</td><td>regulation of protein modification by small protein conjugation or removal</td><td>GO:1903320</td><td>1.92E-06</td><td>0.000117</td><td>5581|83593|51343|23328|3984|11315|5683</td></tr>
<tr><td>49</td><td>cellular response to stimulus</td><td>GO:0051716</td><td>2.41E-06</td><td>0.000143</td><td>5396|83593|10068|100271715|23328|1893|91653|1545|1543|5581|5829|51343|2263|5683|3588|112755|389058|59352|2838|11315|10788|1284|1021|6461|375346|4915|6506|2208|5795|3984|168667|5799</td></tr>
<tr><td>50</td><td>movement of cell or subcellular component</td><td>GO:0006928</td><td>2.60E-06</td><td>0.000151</td><td>91653|1545|1021|64518|56992|5581|23328|5795|59352|2838|196385|4915|2702|168667|1893</td></tr>
</table>

<table>
<tr><td>51</td><td>positive regulation of signaling</td><td>GO:0023056</td><td>3.21E-06</td><td>0.000183</td><td>1545|5396|5581|6461|4915|23328|5795|59352|2263|11315|112755|168667|5683|1893</td></tr>
<tr><td>52</td><td>cardiovascular system development</td><td>GO:0072358</td><td>3.69E-06</td><td>0.000203</td><td>1284|1545|5396|7716|6461|4915|23328|2263|2702|168667|1893</td></tr>
<tr><td>53</td><td>circulatory system development</td><td>GO:0072359</td><td>3.69E-06</td><td>0.000203</td><td>1284|1545|5396|7716|6461|4915|23328|2263|2702|168667|1893</td></tr>
<tr><td>54</td><td>cell projection</td><td>GO:0042995</td><td>3.86E-06</td><td>0.000208</td><td>91653|1021|57465|64518|5829|4915|11252|6506|2702|3984|5795|196385|11315|10788|5799</td></tr>
<tr><td>55</td><td>positive regulation of cellular process</td><td>GO:0048522</td><td>4.08E-06</td><td>0.000216</td><td>5396|127833|83593|23328|1893|91653|1545|1543|5581|51343|2263|5683|2702|59352|11315|10788|3782|2120|1021|112755|6461|4915|5795|3984|168667</td></tr>
<tr><td>56</td><td>response to organic substance</td><td>GO:0010033</td><td>4.37E-06</td><td>0.000227</td><td>1284|1545|1543|5581|5829|10068|4915|6506|7799|23328|1371|3588|389058|5799|2263|11315|168667|5683|1893</td></tr>
<tr><td>57</td><td>regulation of macromolecule metabolic process</td><td>GO:0060255</td><td>5.95E-06</td><td>0.000304</td><td>4915|5396|83593|23328|7799|1893|1545|5581|2208|51343|89932|7553|2263|5683|10643|112755|57491|11315|2120|1021|7716|5795|389058|55657|2005|3984|168667|22887</td></tr>
<tr><td>58</td><td>axon part</td><td>GO:0033267</td><td>8.17E-06</td><td>0.00041</td><td>91653|57465|4915|6506|3984|5799</td></tr>
<tr><td>59</td><td>cellular response to organic substance</td><td>GO:0071310</td><td>8.82E-06</td><td>0.000432</td><td>1284|1545|1543|5581|5829|10068|4915|23328|3588|389058|5799|2263|11315|168667|5683|1893</td></tr>
<tr><td>60</td><td>regulation of cell communication</td><td>GO:0010646</td><td>8.96E-06</td><td>0.000432</td><td>80223|1545|5396|112755|5581|5829|6461|10068|4915|23328|5795|59352|100271715|2263|11315|2702|168667|5683|1893</td></tr>
<tr><td>61</td><td>negative regulation of protein ubiquitination</td><td>GO:0031397</td><td>9.05E-06</td><td>0.000432</td><td>3984|11315|51343|5683|5581</td></tr>
<tr><td>62</td><td>cellular developmental process</td><td>GO:0048869</td><td>9.24E-06</td><td>0.000434</td><td>64518|5396|57465|127833|91653|1543|7716|51343|2263|100526772|1297|2120|84824|1284|1021|6461|4915|11252|2005|3984|168667|54535</td></tr>
<tr><td>63</td><td>cell proliferation</td><td>GO:0008283</td><td>1.01E-05</td><td>0.000466</td><td>5396|2120|1545|1021|1543|83593|56992|51343|5795|2263|4915|100526772|586|168667|1893</td></tr>
<tr><td>64</td><td>regulation of signaling</td><td>GO:0023051</td><td>1.14E-05</td><td>0.000519</td><td>80223|1545|5396|112755|5581|5829|6461|10068|4915|23328|5795|59352|100271715|2263|11315|2702|168667|5683|1893</td></tr>
<tr><td>65</td><td>cellular component organization</td><td>GO:0016043</td><td>1.26E-05</td><td>0.000564</td><td>64518|5396|57465|127833|56992|7799|91653|80222|5581|5829|51343|5192|1299|2263|1297|2702|1545|11315|10788|3782|1284|112755|4915|11252|64983|5795|2005|3984</td></tr>
</table>

<table>
<tr><td>66</td><td>negative regulation of protein modification by small protein conjugation or removal</td><td>GO:1903321</td><td>1.29E-05</td><td>0.000568</td><td>3984|11315|51343|5683|5581</td></tr>
<tr><td>67</td><td>tissue development</td><td>GO:0009888</td><td>1.34E-05</td><td>0.000581</td><td>5396|1284|1545|1021|1543|7716|51343|2702|2263|100526772|168667|1297|5683|1893</td></tr>
<tr><td>68</td><td>regulation of molecular function</td><td>GO:0065009</td><td>1.43E-05</td><td>0.000612</td><td>1545|57465|5581|2208|375346|51343|23328|89932|5795|4915|5799|100271715|3984|2263|11315|10788|5683|1893</td></tr>
<tr><td>69</td><td>negative regulation of molecular function</td><td>GO:0044092</td><td>1.54E-05</td><td>0.000649</td><td>1545|5581|51343|89932|5795|5799|3984|11315|10788|5683|1893</td></tr>
<tr><td>70</td><td>presynaptic process involved in chemical synaptic transmission</td><td>GO:0099531</td><td>1.62E-05</td><td>0.000674</td><td>127833|11315|4915|5799|112755</td></tr>
<tr><td>71</td><td>response to chemical</td><td>GO:0042221</td><td>1.88E-05</td><td>0.00076</td><td>10068|23328|7799|1893|91653|57491|1543|5581|5829|1371|2263|5683|3588|1545|11315|1284|4915|6506|5795|389058|168667|5799</td></tr>
<tr><td>72</td><td>regulation of metabolic process</td><td>GO:0019222</td><td>1.88E-05</td><td>0.00076</td><td>4915|5396|83593|23328|7799|1893|1545|5581|2208|51343|89932|7553|2263|5683|10643|112755|57491|11315|2120|1021|7716|5795|389058|55657|2005|3984|168667|22887</td></tr>
<tr><td>73</td><td>regulation of catalytic activity</td><td>GO:0050790</td><td>1.92E-05</td><td>0.000764</td><td>4915|57465|5581|2208|51343|23328|89932|5795|5799|100271715|3984|2263|11315|10788|5683|1893</td></tr>
<tr><td>74</td><td>cellular component organization or biogenesis</td><td>GO:0071840</td><td>2.02E-05</td><td>0.000795</td><td>64518|5396|57465|127833|56992|7799|91653|80222|5581|5829|51343|5192|1299|2263|1297|2702|1545|11315|10788|3782|1284|112755|4915|11252|64983|5795|2005|3984</td></tr>
<tr><td>75</td><td>regulation of multicellular organismal process</td><td>GO:0051239</td><td>2.15E-05</td><td>0.000836</td><td>1284|80223|1545|1021|5396|5581|127833|4915|10643|23328|3984|2263|11315|2702|168667|5683|1893</td></tr>
<tr><td>76</td><td>secretory granule membrane</td><td>GO:0030667</td><td>2.71E-05</td><td>0.001037</td><td>127833|64518|5799|11336</td></tr>
<tr><td>77</td><td>secretion by cell</td><td>GO:0032940</td><td>2.83E-05</td><td>0.00106</td><td>80223|5581|127833|4915|6506|2702|11336|112755|5799|1893</td></tr>
<tr><td>78</td><td>negative regulation of ubiquitin-protein transferase activity</td><td>GO:0051444</td><td>2.84E-05</td><td>0.00106</td><td>3984|11315|51343|5683</td></tr>
</table>

79	cell differentiation	GO:0030154	2.98E-05	0.001098	91653|4915|1021|1543|57465|7716|127833|6461|51343|54535|1284|84824|2005|3984|2263|100526772|168667|1297|2120|5396
80	axon	GO:0030424	3.19E-05	0.001149	91653|57465|4915|6506|3984|11315|5799
81	regulation of signal transduction	GO:0009966	3.20E-05	0.001149	1545|5396|5581|5829|6461|10068|4915|23328|5795|59352|100271715|2263|11315|112755|168667|5683|1893
82	extracellular matrix	GO:0031012	3.33E-05	0.001169	1284|89797|89932|1299|2263|1297|1893
83	presynapse	GO:0098793	3.33E-05	0.001169	57465|127833|4915|112755|11315|5799
84	negative regulation of cellular process	GO:0048523	3.39E-05	0.001174	5396|83593|10068|1893|80223|57491|5581|51343|89932|2263|5683|10643|1545|11315|2120|1021|4915|11252|5795|2005|3984|168667
85	regulation of response to stimulus	GO:0048583	3.51E-05	0.001203	1545|1021|5396|5581|5829|6461|10068|4915|23328|2208|5795|59352|100271715|3984|2263|11315|112755|168667|5683|1893
86	regulation of cellular component movement	GO:0051270	3.72E-05	0.001258	1545|1021|5581|23328|5795|59352|2702|168667|1893
87	aging	GO:0007568	3.95E-05	0.001322	51343|1021|1543|4915|6506|7799
88	signal transduction	GO:0007165	4.01E-05	0.001327	5396|83593|10068|100271715|23328|1893|91653|1545|5581|5829|2263|5683|3588|112755|59352|2838|11315|10788|1021|6461|375346|4915|2208|5795|3984|168667
89	regulation of neurotransmitter levels	GO:0001505	4.31E-05	0.001412	127833|11315|4915|5799|112755
90	neurotransmitter transport	GO:0006836	5.02E-05	0.001626	127833|11315|4915|5799|112755
91	negative regulation of catalytic activity	GO:0043086	5.82E-05	0.001863	51343|89932|5795|5799|3984|11315|10788|5683|1893
92	locomotion	GO:0040011	6.03E-05	0.00191	91653|1545|1021|64518|5581|23328|5795|59352|2838|4915|168667|1893
93	cell-cell signaling	GO:0007267	6.23E-05	0.00195	5799|80223|5581|127833|4915|6506|2702|59352|2263|11315|112755|5683
94	organic substance metabolic process	GO:0071704	7.10E-05	0.002199	51059|5396|83593|23328|7799|5799|1893|89932|80222|1543|7716|2208|51343|1371|7553|2263|5683|586|1545|2582|2120|10643|112755|389058|11315|57491|4915|1284|1021|5581|64983|5795|55657|2005|3984|9435|168667|22887
95	cell motility	GO:0048870	7.99E-05	0.002422	64518|1545|1021|5581|4915|23328|5795|59352|2838|168667|1893
96	localization of cell	GO:0051674	7.99E-05	0.002422	64518|1545|1021|5581|4915|23328|5795|59352|2838|168667|1893

97	secretion	GO:0046903	8.09E-05	0.002428	80223|5581|127833|4915|6506|2702|11336|112755|5799|1893
98	cellular response to chemical stimulus	GO:0070887	8.24E-05	0.002449	1284|1545|1543|5581|5829|10068|4915|23328|3588|389058|5799|2263|11315|168667|5683|1893
99	sensory organ development	GO:0007423	9.35E-05	0.00275	1545|5396|51343|2263|4915|168667|1543
100	positive regulation of intracellular signal transduction	GO:1902533	0.000101	0.002933	1545|5581|4915|23328|5795|2263|11315|168667|1893
101	cell leading edge	GO:0031252	0.000103	0.002976	1021|5829|11252|6506|5795|10788
102	proteinaceous extracellular matrix	GO:0005578	0.00011	0.003132	1284|89797|89932|1299|1297|1893
103	regulation of ubiquitin-protein transferase activity	GO:0051438	0.000116	0.003275	3984|11315|51343|5683
104	FACIT collagen trimer	GO:0005593	0.000119	0.003302	1299|1297
105	extracellular matrix structural constituent conferring tensile strength	GO:0030020	0.000119	0.003302	1299|1297
106	regulation of cell motility	GO:2000145	0.000134	0.003668	1545|1021|5581|23328|5795|59352|168667|1893
107	regulation of primary metabolic process	GO:0080090	0.000146	0.003976	4915|5396|83593|23328|7799|1893|1545|5581|51343|89932|7553|2263|5683|10643|57491|11315|2120|7716|5795|389058|55657|2005|3984|168667|22887
108	metabolic process	GO:0008152	0.000157	0.004233	51059|5396|83593|23328|7799|5799|1893|89932|80222|1543|7716|2208|51343|1371|7553|2263|5683|586|1545|2582|2120|10643|112755|389058|11315|57491|4915|1284|1021|5581|64983|5795|55657|2005|3984|9435|168667|22887
109	vesicle	GO:0031982	0.000159	0.004254	5799|1284|80223|2582|112755|64518|2208|10068|11252|5683|3588|5795|59352|11336|2263|11315|10788|127833|1893
110	response to stress	GO:0006950	0.000164	0.004337	5396|91653|9435|1021|1543|7716|5829|5581|10068|51343|6506|23328|3588|1545|4915|11315|100526772|5683|1893
111	cellular response to reactive oxygen species	GO:0034614	0.000168	0.0044	5829|10068|1545|11315
112	regulation of cellular	GO:0031323	0.000171	0.004449	4915|5396|83593|23328|7799|1893|1545|5581|51343|89932|7553|2263|5683|10643|57491|11315|2120|7716|5795|389058|55657|2005|39

<table>
<tr><td></td><td>metabolic process</td><td></td><td></td><td></td><td>84|168667|22887</td></tr>
<tr><td>113</td><td>regulation of locomotion</td><td>GO:0040012</td><td>0.000175</td><td>0.004498</td><td>1545|1021|5581|23328|5795|59352|168667|1893</td></tr>
<tr><td>114</td><td>organelle membrane</td><td>GO:0031090</td><td>0.000183</td><td>0.004671</td><td>64518|80223|4915|127833|51343|11252|64983|5192|1371|23274|59352|11336|11315|9435|5799</td></tr>
<tr><td>115</td><td>negative regulation of cellular protein metabolic process</td><td>GO:0032269</td><td>0.00019</td><td>0.004758</td><td>5581|51343|10643|89932|5795|3984|11315|5683|1893</td></tr>
<tr><td>116</td><td>intracellular organelle lumen</td><td>GO:0070013</td><td>0.000193</td><td>0.004758</td><td>2120|1284|80222|1021|5396|7716|5829|51343|11252|64983|89797|1371|1299|5799|2005|3984|2263|11315|1297|5683|1893</td></tr>
<tr><td>117</td><td>membrane-enclosed lumen</td><td>GO:0031974</td><td>0.000194</td><td>0.004758</td><td>2120|1284|80222|1021|5396|7716|5829|51343|11252|64983|89797|1371|1299|5799|2005|3984|2263|11315|1297|5683|1893</td></tr>
<tr><td>118</td><td>organelle lumen</td><td>GO:0043233</td><td>0.000194</td><td>0.004758</td><td>2120|1284|80222|1021|5396|7716|5829|51343|11252|64983|89797|1371|1299|5799|2005|3984|2263|11315|1297|5683|1893</td></tr>
<tr><td>119</td><td>animal organ development</td><td>GO:0048513</td><td>0.000194</td><td>0.004758</td><td>5396|2120|1545|1021|1543|51343|6506|2702|1299|389058|2263|4915|100526772|168667|1297|5683|1893</td></tr>
<tr><td>120</td><td>macromolecule metabolic process</td><td>GO:0043170</td><td>0.0002</td><td>0.004855</td><td>4915|5396|83593|23328|7799|5799|1893|80222|7716|2208|51343|89932|7553|2263|5683|1545|2120|10643|112755|389058|11315|57491|
1284|1021|5581|64983|5795|55657|2005|3984|9435|168667|22887</td></tr>
<tr><td>121</td><td>cytosol</td><td>GO:0005829</td><td>0.000204</td><td>0.0049</td><td>586|4915|1021|2582|5581|5829|6461|56992|51343|11252|10643|112755|127833|11336|3984|11315|10788|5683</td></tr>
<tr><td>122</td><td>neurotransmitter secretion</td><td>GO:0007269</td><td>0.000222</td><td>0.005264</td><td>127833|4915|5799|112755</td></tr>
<tr><td>123</td><td>signal release from synapse</td><td>GO:0099643</td><td>0.000222</td><td>0.005264</td><td>127833|4915|5799|112755</td></tr>
<tr><td>124</td><td>omega-hydroxylase P450 pathway</td><td>GO:0097267</td><td>0.000233</td><td>0.005385</td><td>1545|1543</td></tr>
<tr><td>125</td><td>synaptic signaling</td><td>GO:0099536</td><td>0.000237</td><td>0.005385</td><td>5581|127833|4915|6506|112755|11315|5799</td></tr>
<tr><td>126</td><td>anterograde trans-synaptic signaling</td><td>GO:0098916</td><td>0.000237</td><td>0.005385</td><td>5581|127833|4915|6506|112755|11315|5799</td></tr>
<tr><td>127</td><td>chemical synaptic transmission</td><td>GO:0007268</td><td>0.000237</td><td>0.005385</td><td>5581|127833|4915|6506|112755|11315|5799</td></tr>
<tr><td>128</td><td>trans-synaptic signaling</td><td>GO:0099537</td><td>0.000237</td><td>0.005385</td><td>5581|127833|4915|6506|112755|11315|5799</td></tr>
<tr><td>129</td><td>negative regulation of macromolecule metabolic process</td><td>GO:0010605</td><td>0.00025</td><td>0.005647</td><td>2120|57491|5396|5581|51343|10643|89932|5795|2005|3984|2263|11315|5683|1893</td></tr>
</table>

130	response to oxygen-containing compound	GO:1901700	0.000256	0.005694	1545|1543|5581|5829|10068|4915|6506|23328|7799|11315|5799
131	regulation of anatomical structure morphogenesis	GO:0022603	0.000256	0.005694	1284|1545|127833|4915|23328|3984|2263|5683|1893
132	signal release	GO:0023061	0.000269	0.005925	80223|5581|127833|4915|112755|5799
133	negative regulation of cellular metabolic process	GO:0031324	0.000275	0.006014	2120|57491|5396|5581|51343|10643|89932|5795|2005|3984|2263|11315|5683|1893
134	regulation of cellular protein metabolic process	GO:0032268	0.000281	0.006102	4915|5581|83593|51343|10643|23328|89932|5795|3984|2263|11315|168667|5683|1893
135	negative regulation of protein metabolic process	GO:0051248	0.000289	0.006244	5581|51343|10643|89932|5795|3984|11315|5683|1893
136	cytoplasmic vesicle part	GO:0044433	0.000292	0.00626	64518|80223|127833|11252|11336|5799|1893
137	intracellular signal transduction	GO:0035556	0.000298	0.006301	1545|5581|83593|375346|4915|23328|5795|100271715|3984|2263|11315|10788|168667|5683|1893
138	gene expression	GO:0010467	0.000299	0.006301	5396|7799|1893|80222|7716|7553|2263|5683|1284|10643|112755|1545|11315|57491|2120|1021|4915|64983|389058|55657|2005|168667|22887
139	membrane-bounded vesicle	GO:0031988	0.000304	0.006361	5799|1284|80223|2582|64518|2208|10068|11252|112755|3588|5795|5683|11336|2263|11315|10788|127833|1893
140	regulation of gene expression	GO:0010468	0.000347	0.007218	2120|1545|57491|1021|5396|7716|4915|10643|112755|7799|389058|55657|22887|2005|7553|2263|11315|168667|5683|1893
141	neuron part	GO:0097458	0.000357	0.00738	3782|91653|57465|127833|4915|6506|112755|3984|11315|5799
142	single-organism transport	GO:0044765	0.000374	0.007669	3782|80223|112755|5581|127833|375346|4915|6506|2702|11336|3984|11315|5192|5799|1893
143	toxin metabolic process	GO:0009404	0.000384	0.007757	1545|1543
144	response to insecticide	GO:0017085	0.000384	0.007757	1371|1543
145	cellular metabolic process	GO:0044237	0.000386	0.007757	51059|5396|83593|23328|7799|5799|1893|89932|80222|1543|7716|51343|1371|7553|2263|5683|586|1545|2120|10643|389058|11315|57491|4915|1284|1021|5581|64983|5795|55657|2005|3984|9435|168667|22887
146	microtubule cytoskeleton	GO:0015630	0.00039	0.007781	64518|1021|5829|83593|56992|112755|196385|10788|54535

147	terminal bouton	GO:0043195	0.000396	0.007848	4915|5799|57465
148	cell periphery	GO:0071944	0.000428	0.008412	127833|2838|91653|5581|5829|2263|28316|3588|2702|59352|11336|11315|112755|3782|6461|375346|4915|11252|6506|2208|5795|5799
149	regulation of hydrolase activity	GO:0051336	0.000433	0.008457	57465|5581|4915|89932|100271715|2263|11315|10788|5799|1893
150	secretory vesicle	GO:0099503	0.000463	0.008986	64518|127833|112755|11336|5799|1893
151	heterocyclic compound binding	GO:1901363	0.000466	0.008992	4915|5396|56992|7799|196385|80222|1543|7716|89797|7553|2263|10643|1545|11315|57491|2120|1021|5581|64983|389058|55657|2005|3984|22887
152	membrane part	GO:0044425	0.000492	0.009427	387914|91653|1545|1543|2208|5192|127833|2263|100526772|28316|3588|2702|59352|2838|11315|112755|3782|375346|4915|11252|6506|5795|148808|9435|154467|5799
153	molecular function regulator	GO:0098772	0.0005	0.009525	57465|5581|375346|51343|89932|100271715|2263|11315|10788|10068
154	whole membrane	GO:0098805	0.000509	0.009627	64518|80223|127833|4915|11252|5192|23274|11336|11315|5799
155	regulation of cell migration	GO:0030334	0.000516	0.009691	1545|5581|23328|5795|59352|168667|1893
156	regulation of protein metabolic process	GO:0051246	0.000524	0.009789	4915|5581|83593|51343|10643|23328|89932|5795|3984|2263|11315|168667|5683|1893
157	cytoplasmic vesicle membrane	GO:0030659	0.000534	0.009903	64518|80223|127833|11252|11336|5799
158	skeletal system development	GO:0001501	0.000557	0.010273	5396|2702|389058|2263|100526772|1297
159	organic cyclic compound binding	GO:0097159	0.000571	0.010361	4915|5396|56992|7799|196385|80222|1543|7716|89797|7553|2263|10643|1545|11315|57491|2120|1021|5581|64983|389058|55657|2005|3984|22887
160	endoplasmic reticulum	GO:0005783	0.000572	0.010361	1284|1545|1543|5581|387914|375346|4915|1299|11315|1297|5799
161	negative regulation of metabolic process	GO:0009892	0.000573	0.010361	2120|57491|5396|5581|51343|10643|89932|5795|2005|3984|2263|11315|5683|1893
162	anatomical structure formation involved in morphogenesis	GO:0048646	0.000607	0.010915	1284|1545|7716|6461|23328|2263|2702|168667|1893
163	positive regulation of protein	GO:0031401	0.000615	0.010919	4915|5581|83593|51343|23328|2263|11315|168667|5683

	modification process				
164	extracellular matrix structural constituent	GO:0005201	0.000615	0.010919	1284|1299|1297
165	regulation of protein modification process	GO:0031399	0.000627	0.010996	4915|5581|83593|51343|23328|5795|3984|2263|11315|168667|5683
166	extracellular matrix organization	GO:0030198	0.00063	0.010996	1284|11315|1299|1297|1545
167	extracellular region part	GO:0044421	0.000633	0.010996	1284|2582|64518|2208|10068|11252|89797|89932|3588|5795|1299|2263|11315|10788|168667|1297|5683|1893
168	cellular response to hydrogen peroxide	GO:0070301	0.000638	0.010996	10068|1545|11315
169	extracellular structure organization	GO:0043062	0.000639	0.010996	1284|11315|1299|1297|1545
170	calcium-mediated signaling using intracellular calcium source	GO:0035584	0.000642	0.010996	375346|4915
171	vesicle membrane	GO:0012506	0.000653	0.011063	64518|80223|127833|11252|11336|5799
172	cellular macromolecule metabolic process	GO:0044260	0.000653	0.011063	4915|5396|83593|23328|7799|5799|1893|80222|5581|51343|89932|7553|2263|5683|1545|2120|10643|389058|11315|57491|1284|1021|7716|64983|5795|55657|2005|3984|168667|22887
173	cellular response to oxygen-containing compound	GO:1901701	0.000658	0.011073	1545|5581|5829|10068|4915|23328|11315|5799
174	regulation of developmental process	GO:0050793	0.000686	0.011483	1284|1545|1021|5396|91653|127833|51343|23328|3984|2263|4915|5683|1893
175	cell projection part	GO:0044463	0.000701	0.011663	91653|57465|4915|11252|6506|5795|3984|5799
176	regulation of multicellular organismal development	GO:2000026	0.000709	0.011736	1284|1545|1021|5396|127833|4915|23328|3984|2263|5683|1893
177	negative regulation of cell	GO:0090344	0.000716	0.01174	51343|1021

	aging				
178	transport	GO:0006810	0.000718	0.01174	3782|80223|112755|5581|127833|375346|4915|11252|10643|2702|56992|6506|11336|3984|148808|11315|5192|54535|5799|1893
179	positive regulation of multicellular organismal process	GO:0051240	0.000723	0.011758	1545|5581|127833|4915|23328|3984|2263|11315|2702|1893
180	cytoplasmic, membrane-bounded vesicle	GO:0016023	0.00073	0.011816	64518|80223|127833|11252|112755|11336|2263|5799|1893
181	epoxygenase P450 pathway	GO:0019373	0.000795	0.01272	1545|1543
182	bounding membrane of organelle	GO:0098588	0.000812	0.012741	64518|80223|127833|4915|11252|5192|23274|59352|11336|9435|5799
183	cell migration	GO:0016477	0.000814	0.012741	1545|5581|4915|23328|5795|59352|2838|168667|1893
184	single-organism localization	GO:1902578	0.000814	0.012741	3782|80223|112755|5581|127833|375346|4915|6506|2702|11336|3984|11315|5192|5799|1893
185	primary metabolic process	GO:0044238	0.000831	0.01294	51059|5396|83593|23328|7799|5799|1893|80222|1543|7716|51343|89932|7553|2263|5683|1545|2582|2120|10643|389058|11315|57491|4915|1284|1021|5581|64983|5795|55657|2005|3984|9435|168667|22887
186	reactive oxygen species biosynthetic process	GO:1903409	0.000842	0.01304	11315|1545|1543
187	axonal growth cone	GO:0044295	0.000877	0.01352	3984|91653
188	integral component of membrane	GO:0016021	0.000884	0.013544	387914|91653|2208|5192|127833|2263|100526772|28316|3588|112755|59352|2838|2702|3782|375346|4915|6506|5795|148808|9435|154467|5799
189	endoplasmic reticulum lumen	GO:0005788	0.000894	0.01363	1284|1299|1297|5799
190	synapse	GO:0045202	0.0009	0.013653	57465|127833|4915|112755|2263|11315|5799
191	negative regulation of transferase activity	GO:0051348	0.000909	0.013654	3984|5795|51343|11315|5683
192	organic substance transport	GO:0071702	0.00091	0.013654	3782|80223|112755|5581|375346|4915|10643|2702|6506|11336|11315|5192|54535|5799
193	collagen trimer	GO:0005581	0.000928	0.013856	1284|1299|1297

<table>
<tr><td></td><td></td><td></td><td></td><td></td><td></td></tr>
<tr><td>194</td><td>regulation of cellular component biogenesis</td><td>GO:0044087</td><td>0.000951</td><td>0.014054</td><td>5581|4915|112755|5795|3984|11315|10788</td></tr>
<tr><td>195</td><td>organic substance biosynthetic process</td><td>GO:1901576</td><td>0.000958</td><td>0.01409</td><td>5396|7799|1893|80222|1543|7716|2208|1371|7553|2263|586|1284|10643|1545|11315|57491|2120|64983|389058|55657|2005|9435|168667|22887</td></tr>
<tr><td>196</td><td>secretory granule</td><td>GO:0030141</td><td>0.000967</td><td>0.014152</td><td>127833|64518|11336|5799|1893</td></tr>
<tr><td>197</td><td>ion binding</td><td>GO:0043167</td><td>0.000991</td><td>0.014353</td><td>89932|1545|1543|7716|5829|83593|5581|11252|28316|7799|2208|389058|55657|3984|11315|5192|1297|127833|7553</td></tr>
<tr><td>198</td><td>establishment of localization</td><td>GO:0051234</td><td>0.00102</td><td>0.014653</td><td>3782|80223|112755|5581|127833|375346|4915|11252|10643|2702|56992|6506|11336|3984|148808|11315|5192|54535|5799|1893</td></tr>
<tr><td>199</td><td>enzyme binding</td><td>GO:0019899</td><td>0.001023</td><td>0.014653</td><td>80223|1543|112755|5581|5829|4915|23328|5795|11315|10788|1893</td></tr>
<tr><td>200</td><td>cell projection organization</td><td>GO:0030030</td><td>0.001027</td><td>0.014653</td><td>91653|5396|57465|64518|127833|4915|11252|3984|2263</td></tr>
<tr><td>201</td><td>protein complex</td><td>GO:0043234</td><td>0.00106</td><td>0.015046</td><td>1284|10788|1021|112755|5829|56992|51343|23328|3588|1299|5799|11336|196385|11315|2702|1297|5683|4915</td></tr>
<tr><td>202</td><td>response to toxic substance</td><td>GO:0009636</td><td>0.001067</td><td>0.015046</td><td>1371|11315|1545|1543</td></tr>
<tr><td>203</td><td>epithelial cell differentiation</td><td>GO:0030855</td><td>0.001074</td><td>0.015046</td><td>1021|1543|7716|51343|2263|168667</td></tr>
<tr><td>204</td><td>protein ubiquitination</td><td>GO:0016567</td><td>0.001075</td><td>0.015046</td><td>5581|83593|51343|23328|3984|11315|5683</td></tr>
<tr><td>205</td><td>regulation of angiogenesis</td><td>GO:0045765</td><td>0.001104</td><td>0.015389</td><td>1284|1893|1545|23328</td></tr>
<tr><td>206</td><td>macromolecule biosynthetic process</td><td>GO:0009059</td><td>0.001154</td><td>0.015962</td><td>2120|1284|80222|5396|1545|7716|2208|64983|10643|7799|57491|55657|389058|2005|7553|2263|11315|9435|168667|22887|1893</td></tr>
<tr><td>207</td><td>response to reactive oxygen species</td><td>GO:0000302</td><td>0.001162</td><td>0.015962</td><td>5829|10068|1545|11315</td></tr>
<tr><td>208</td><td>regulation of transport</td><td>GO:0051049</td><td>0.001178</td><td>0.016098</td><td>3782|80223|5581|127833|375346|4915|11252|6506|2702|11315|112755</td></tr>
<tr><td>209</td><td>biosynthetic process</td><td>GO:0009058</td><td>0.0012</td><td>0.016263</td><td>5396|7799|1893|80222|1543|7716|2208|1371|7553|2263|586|1284|10643|1545|11315|57491|2120|64983|389058|55657|2005|9435|168667|22887</td></tr>
<tr><td>210</td><td>intrinsic component of membrane</td><td>GO:0031224</td><td>0.001201</td><td>0.016263</td><td>387914|91653|2208|5192|127833|2263|100526772|28316|3588|112755|59352|2838|2702|3782|375346|4915|6506|5795|148808|9435|154467|5799</td></tr>
<tr><td>211</td><td>epithelial cell migration</td><td>GO:0010631</td><td>0.001222</td><td>0.016471</td><td>1545|5581|168667|23328</td></tr>
<tr><td>212</td><td>cellular response to oxidative stress</td><td>GO:0034599</td><td>0.001242</td><td>0.016654</td><td>5829|10068|1545|11315</td></tr>
</table>

213	aromatase activity	GO:0070330	0.001247	0.016654	1545|1543
214	negative regulation of protein modification process	GO:0031400	0.001265	0.016777	5581|51343|5795|3984|11315|5683
215	positive regulation of molecular function	GO:0044093	0.00127	0.016777	57465|5581|2208|375346|51343|23328|100271715|2263|11315|10788|5683
216	cell projection morphogenesis	GO:0048858	0.001275	0.016777	91653|64518|127833|4915|11252|3984|2263
217	epithelium migration	GO:0090132	0.001284	0.016777	1545|5581|168667|23328
218	cytoplasmic vesicle	GO:0031410	0.00129	0.016777	64518|80223|127833|11252|112755|11336|2263|5799|1893
219	positive regulation of catalytic activity	GO:0043085	0.001291	0.016777	57465|5581|2208|51343|23328|100271715|2263|11315|10788|5683
220	intracellular vesicle	GO:0097708	0.001304	0.016875	64518|80223|127833|11252|112755|11336|2263|5799|1893
221	protein localization	GO:0008104	0.001324	0.017019	3782|80223|112755|5581|83593|375346|11252|2702|11336|11315|5192|54535|5799
222	positive regulation of cell-substrate adhesion	GO:0010811	0.001327	0.017019	5795|1021|5581
223	exocytosis	GO:0006887	0.001376	0.017539	127833|80223|11336|112755|1893
224	cellular localization	GO:0051641	0.001379	0.017539	3782|5581|127833|83593|375346|4915|11252|5192|11336|11315|112755|54535|5799
225	protein transport	GO:0015031	0.0014	0.017721	3782|80223|112755|5581|375346|2702|11336|11315|5192|54535|5799
226	tissue migration	GO:0090130	0.001414	0.017821	1545|5581|168667|23328
227	response to external stimulus	GO:0009605	0.001431	0.017965	91653|1021|1543|5581|3588|10068|4915|6506|23328|5795|11315|5683
228	negative regulation of hydrolase activity	GO:0051346	0.001455	0.018188	89932|11315|10788|5799|1893
229	cell part morphogenesis	GO:0032990	0.001472	0.018315	91653|64518|127833|4915|11252|3984|2263
230	cellular protein metabolic process	GO:0044267	0.001484	0.01833	89932|80222|1021|5581|83593|51343|64983|23328|7799|5795|4915|5799|10643|2005|3984|2263|11315|168667|5683|1893
231	immune system process	GO:0002376	0.001486	0.01833	2120|1021|5581|2208|10068|3588|5795|2838|3984|2263|56992|5683|1893

232	neuron projection development	GO:0031175	0.001503	0.01847	91653|5396|57465|127833|4915|3984|2263
233	metal ion binding	GO:0046872	0.001519	0.018521	89932|1545|1543|7716|5829|83593|5581|28316|7799|2208|389058|55657|3984|11315|5192|1297|127833|7553
234	synapse part	GO:0044456	0.001532	0.018521	57465|127833|4915|112755|11315|5799
235	microtubule	GO:0005874	0.001538	0.018521	196385|83593|56992|10788|64518
236	single-organism catabolic process	GO:0044712	0.001546	0.018521	586|1284|1545|1543|5581|11315|2582
237	macromolecule localization	GO:0033036	0.001549	0.018521	3782|80223|112755|5581|83593|375346|11252|10643|2702|11336|11315|5192|54535|5799
238	regulation of vasculature development	GO:1901342	0.001553	0.018521	1284|1893|1545|23328
239	response to xenobiotic stimulus	GO:0009410	0.001561	0.018521	1545|57491|1543
240	epithelium development	GO:0060429	0.001563	0.018521	1021|1543|7716|51343|2702|2263|168667|5683
241	oxidoreductase activity, acting on paired donors, with incorporation or reduction of molecular oxygen, reduced flavin or flavoprotein as one donor, and incorporation of one atom of oxygen	GO:0016712	0.001565	0.018521	1545|1543
242	integral component of plasma membrane	GO:0005887	0.001578	0.018601	91653|2208|4915|2702|3588|5795|59352|2838|2263|5799
243	regulation of biological quality	GO:0065008	0.001599	0.018721	80223|1545|1021|112755|5581|2208|4915|89797|2702|127833|5795|5799|3984|11315|10788|5683
244	regulation of cellular localization	GO:0060341	0.001601	0.018721	3782|5581|83593|375346|4915|112755|11315

245	cell cortex	GO:0005938	0.001676	0.01947	5829|2263|375346|11336
246	positive regulation of mesenchymal cell proliferation	GO:0002053	0.001678	0.01947	2263|5396
247	cation binding	GO:0043169	0.001692	0.019552	89932|1545|1543|7716|5829|83593|5581|28316|7799|2208|389058|55657|3984|11315|5192|1297|127833|7553
248	endothelium development	GO:0003158	0.00173	0.019917	2702|168667|7716
249	cellular biosynthetic process	GO:0044249	0.001772	0.020246	5396|7799|1893|80222|1543|7716|1371|7553|2263|586|1284|10643|1545|11315|57491|2120|64983|389058|55657|2005|9435|168667|22887
250	cellular nitrogen compound biosynthetic process	GO:0044271	0.001773	0.020246	2120|7799|80222|5396|1545|7716|64983|10643|1371|1284|55657|389058|2005|7553|2263|11315|168667|57491|22887|1893
251	ventricular septum morphogenesis	GO:0060412	0.001796	0.020346	2263|2702
252	positive regulation of monooxygenase activity	GO:0032770	0.001796	0.020346	2208|11315
253	kinase binding	GO:0019900	0.001824	0.020589	5829|4915|112755|5795|11315|23328
254	regulation of secretion by cell	GO:1903530	0.0019	0.021359	80223|5581|127833|4915|112755|2702
255	response to arsenic-containing substance	GO:0046685	0.001917	0.02147	1371|1543
256	nitrogen compound metabolic process	GO:0006807	0.001957	0.02183	5396|7799|1893|80222|1543|7716|51343|1371|7553|2263|5683|586|1284|10643|1545|11315|57491|2120|64983|389058|55657|2005|9435|168667|22887
257	positive regulation of cellular component biogenesis	GO:0044089	0.001981	0.022016	3984|5795|10788|4915|5581
258	smoothened signaling pathway	GO:0007224	0.002006	0.022123	91653|5396|2263

259	positive regulation of synaptic transmission	GO:0050806	0.002006	0.022123	4915|112755|5581
260	intrinsic component of plasma membrane	GO:0031226	0.002049	0.022486	91653|2208|4915|2702|3588|5795|59352|2838|2263|5799
261	axon terminus	GO:0043679	0.002054	0.022486	4915|5799|57465
262	plasma membrane	GO:0005886	0.002078	0.022658	3782|91653|5581|5829|6461|4915|11252|6506|2702|2208|5795|5799|59352|2838|2263|11315|112755|3588|28316|127833
263	MAPK cascade	GO:0000165	0.002096	0.022658	5581|4915|23328|5795|2263|168667|5683
264	catalytic activity	GO:0003824	0.002097	0.022658	56992|7799|196385|1371|80222|1543|5581|89797|89932|2263|5683|586|2582|1545|11315|1021|4915|51059|5795|3984|9435|5799
265	intracellular non-membrane-bounded organelle	GO:0043232	0.002109	0.022658	64518|1021|5396|7716|5829|83593|56992|5581|11252|64983|112755|2120|196385|11315|10788|54535|5683
266	non-membrane-bounded organelle	GO:0043228	0.002109	0.022658	64518|1021|5396|7716|5829|83593|56992|5581|11252|64983|112755|2120|196385|11315|10788|54535|5683
267	positive regulation of canonical Wnt signaling pathway	GO:0090263	0.002153	0.022964	2263|59352|5683
268	amine metabolic process	GO:0009308	0.002153	0.022964	11315|1543|5683
269	multicellular organism aging	GO:0010259	0.002171	0.02307	7799|6506
270	SNARE binding	GO:0000149	0.002203	0.023248	127833|112755|11336
271	regulation of cellular component organization	GO:0051128	0.002203	0.023248	3782|4915|5396|5581|127833|51343|11252|112755|5795|3984|11315|10788
272	regulation of protein kinase B signaling	GO:0051896	0.002255	0.023702	5795|4915|11315
273	positive regulation of angiogenesis	GO:0045766	0.002307	0.024162	1545|1893|23328
274	protein modification by small protein conjugation	GO:0032446	0.002384	0.024884	5581|83593|51343|23328|3984|11315|5683

275	vesicle-mediated transport	GO:0016192	0.002434	0.025282	80223|5581|127833|56992|11252|112755|11336|3984|1893
276	regulation of cell aging	GO:0090342	0.00244	0.025282	51343|1021
277	regulation of reproductive process	GO:2000241	0.002467	0.025381	2263|51343|64518
278	response to hydrogen peroxide	GO:0042542	0.002467	0.025381	10068|1545|11315
279	regulation of intracellular signal transduction	GO:1902531	0.002475	0.025381	1545|5581|4915|23328|5795|100271715|2263|11315|168667|1893
280	nucleus	GO:0005634	0.0025	0.025541	64518|5396|83593|7799|5581|5829|51343|89797|7553|2263|100526772|5683|10643|112755|389058|11315|2120|1021|7716|11252|55657|2005|3984|54535|22887
281	synaptic vesicle	GO:0008021	0.002578	0.026174	127833|5799|112755
282	regulation of mesenchymal cell proliferation	GO:0010464	0.00258	0.026174	2263|5396
283	positive regulation of cellular protein metabolic process	GO:0032270	0.002627	0.02656	4915|5581|83593|51343|23328|2263|11315|168667|5683
284	signal transduction by protein phosphorylation	GO:0023014	0.002636	0.026562	5581|4915|23328|5795|2263|168667|5683
285	regulation of MAPK cascade	GO:0043408	0.002651	0.026624	5581|4915|23328|5795|2263|168667
286	regulated exocytosis	GO:0045055	0.002661	0.026631	127833|80223|112755|1893
287	macromolecular complex	GO:0032991	0.002684	0.02677	1284|10788|1021|112755|5829|56992|51343|64983|23328|3588|1299|5799|11336|196385|11315|2702|1297|5683|4915
288	response to iron ion	GO:0010039	0.002723	0.026958	1371|1543
289	porphyrin-containing compound metabolic process	GO:0006778	0.002723	0.026958	1371|1543
290	establishment of protein localization	GO:0045184	0.002745	0.026958	3782|80223|112755|5581|375346|2702|11336|11315|5192|54535|5799

291	neuron projection terminus	GO:0044306	0.00275	0.026958	4915|5799|57465
292	neuron-neuron synaptic transmission	GO:0007270	0.00275	0.026958	11315|4915|5581
293	cytoskeleton	GO:0005856	0.002809	0.027443	64518|1021|5581|5829|83593|56992|11252|112755|196385|10788|54535
294	positive regulation of developmental process	GO:0051094	0.002818	0.027443	91653|1545|127833|4915|23328|3984|2263|1893
295	positive regulation of cellular component organization	GO:0051130	0.002863	0.027773	3782|5581|127833|4915|5795|3984|11315|10788
296	T cell migration	GO:0072678	0.002871	0.027773	1893|2838
297	regulation of secretion	GO:0051046	0.002896	0.027928	80223|5581|127833|4915|112755|2702
298	leading edge membrane	GO:0031256	0.002928	0.028141	5795|11252|6506
299	endoplasmic reticulum part	GO:0044432	0.002985	0.028595	1284|1545|1543|387914|375346|1299|1297|5799
300	organelle organization	GO:0006996	0.003005	0.028693	3782|91653|80222|112755|5581|127833|56992|51343|11252|64983|5192|7799|2005|3984|11315|10788
301	regulation of GTPase activity	GO:0043087	0.003025	0.028763	57465|4915|100271715|2263|10788|5799
302	positive regulation of vasculature development	GO:1904018	0.003051	0.028763	1545|1893|23328
303	response to other organism	GO:0051707	0.003052	0.028763	1021|1543|5581|3588|10068|23328|5683
304	response to external biotic stimulus	GO:0043207	0.003052	0.028763	1021|1543|5581|3588|10068|23328|5683
305	camera-type eye development	GO:0043010	0.003179	0.029862	1545|51343|1543|4915
306	positive regulation of MAPK cascade	GO:0043410	0.003195	0.029915	2263|4915|5581|168667|23328
307	regulation of macromolecule	GO:0010556	0.003256	0.030312	2120|1545|57491|5396|7716|2208|10643|7799|389058|55657|2005|7553|2263|11315|168667|22887|1893

	biosynthetic process				
308	cytoplasmic region	GO:0099568	0.003258	0.030312	5829|2263|375346|11336
309	transcription, DNA-templated	GO:0006351	0.003388	0.031422	2120|1284|57491|5396|1545|7716|7799|389058|55657|2005|7553|2263|11315|168667|22887|1893
310	organic acid metabolic process	GO:0006082	0.003416	0.031484	586|1545|1543|5581|80222|11315|5683
311	ATP binding	GO:0005524	0.003417	0.031484	80222|1021|5581|56992|4915|89797|3984|196385|2263
312	exocytic vesicle	GO:0070382	0.003439	0.031591	127833|5799|112755
313	positive regulation of cell-matrix adhesion	GO:0001954	0.003497	0.031924	5795|1021
314	nucleic acid-templated transcription	GO:0097659	0.003518	0.032016	2120|1284|57491|5396|1545|7716|7799|389058|55657|2005|7553|2263|11315|168667|22887|1893
315	neuron development	GO:0048666	0.003558	0.032274	91653|5396|57465|127833|4915|3984|2263
316	immune response-activating cell surface receptor signaling pathway	GO:0002429	0.003588	0.032447	3984|5795|5683|5581
317	regulation of protein localization	GO:0032880	0.003599	0.032447	3782|80223|5581|83593|375346|2702|11315
318	cytoskeletal part	GO:0044430	0.003621	0.03254	64518|1021|5829|83593|56992|112755|196385|10788|54535
319	growth cone	GO:0030426	0.003644	0.032547	3984|91653|4915
320	microtubule associated complex	GO:0005875	0.003644	0.032547	196385|5829|56992
321	response to biotic stimulus	GO:0009607	0.003768	0.033342	1021|1543|5581|3588|10068|23328|5683
322	limb morphogenesis	GO:0035108	0.003784	0.033342	2263|5396|2702
323	protein kinase B signaling	GO:0043491	0.003784	0.033342	5795|4915|11315
324	appendage morphogenesis	GO:0035107	0.003784	0.033342	2263|5396|2702

325	neuron projection	GO:0043005	0.00379	0.033342	91653|57465|4915|6506|3984|11315|5799
326	mesenchymal cell proliferation	GO:0010463	0.003832	0.033611	2263|5396
327	positive regulation of protein metabolic process	GO:0051247	0.003885	0.033961	4915|5581|83593|51343|23328|2263|11315|168667|5683
328	cell-substrate adhesion	GO:0031589	0.003895	0.033961	5829|5795|1021|5581
329	site of polarized growth	GO:0030427	0.004001	0.034503	3984|91653|4915
330	glutamate secretion	GO:0014047	0.004005	0.034503	4915|6506
331	regulation of endocrine process	GO:0044060	0.004005	0.034503	80223|2702
332	negative regulation of GTPase activity	GO:0034260	0.004005	0.034503	10788|5799
333	adenyl ribonucleotide binding	GO:0032559	0.004022	0.034552	80222|1021|5581|56992|4915|89797|3984|196385|2263
334	regulation of transcription from RNA polymerase II promoter	GO:0006357	0.004054	0.034718	2120|57491|5396|7716|2005|2263|11315|168667|22887|1893
335	extracellular region	GO:0005576	0.004103	0.034941	1284|2582|64518|2208|10068|11252|89797|89932|3588|5795|1299|2263|11315|10788|168667|1297|5683|1893
336	structural molecule activity	GO:0005198	0.004104	0.034941	1284|64983|23328|1371|1299|1297
337	organ morphogenesis	GO:0009887	0.004148	0.035129	5396|4915|2702|389058|2263|1297|5683
338	endothelial cell migration	GO:0043542	0.00415	0.035129	1545|168667|23328
339	regulation of cell proliferation	GO:0042127	0.004182	0.035257	1545|1021|5396|83593|51343|5795|2263|4915|1893
340	adenyl nucleotide binding	GO:0030554	0.004218	0.035257	80222|1021|5581|56992|4915|89797|3984|196385|2263
341	regulation of actin filament-based process	GO:0032970	0.00422	0.035257	3984|5581|10788|2702

342	positive regulation of Wnt signaling pathway	GO:0030177	0.004225	0.035257	2263|59352|5683
343	response to transition metal nanoparticle	GO:1990267	0.004225	0.035257	1371|11315|1543
344	cell surface	GO:0009986	0.004299	0.035764	2208|4915|6506|5795|2263|10788
345	response to inorganic substance	GO:0010035	0.004315	0.035774	1371|10068|1545|11315|1543
346	hydrogen peroxide metabolic process	GO:0042743	0.004361	0.035774	11315|1543
347	intrinsic apoptotic signaling pathway in response to oxidative stress	GO:0008631	0.004361	0.035774	11315|1545
348	receptor regulator activity	GO:0030545	0.004361	0.035774	10068|5581
349	positive regulation of receptor activity	GO:2000273	0.004361	0.035774	11315|5581
350	cellular macromolecule biosynthetic process	GO:0034645	0.004447	0.036358	2120|1284|80222|5396|1545|7716|64983|10643|7799|57491|55657|389058|2005|7553|2263|11315|168667|22887|1893
351	positive regulation of developmental growth	GO:0048639	0.004457	0.036358	3984|2263|127833
352	protein metabolic process	GO:0019538	0.004481	0.03645	89932|80222|1021|5581|83593|51343|64983|23328|7799|5795|4915|5799|10643|2005|3984|2263|11315|168667|5683|1893
353	ruffle	GO:0001726	0.004536	0.03666	1021|5795|11252
354	embryonic cranial skeleton morphogenesis	GO:0048701	0.004545	0.03666	2263|5396
355	positive regulation of oxidoreductase activity	GO:0051353	0.004545	0.03666	2208|11315
356	ameboidal-type cell	GO:0001667	0.004562	0.036694	1545|5581|168667|23328

	migration				
357	regulation of protein transport	GO:0051223	0.004709	0.037774	3782|80223|5581|375346|2702|11315
358	glandular epithelial cell differentiation	GO:0002067	0.004732	0.037854	2263|1021
359	cellular response to stress	GO:0033554	0.004765	0.038014	91653|1545|1021|5396|5581|5829|10068|51343|23328|11315
360	positive regulation of macromolecule metabolic process	GO:0010604	0.004812	0.038285	2120|4915|1021|5396|5581|2208|83593|51343|23328|2263|11315|168667|5683
361	RNA biosynthetic process	GO:0032774	0.004825	0.038286	2120|1284|57491|5396|1545|7716|7799|389058|55657|2005|7553|2263|11315|168667|22887|1893
362	small molecule catabolic process	GO:0044282	0.004869	0.038531	586|11315|2582|1543
363	protein phosphorylation	GO:0006468	0.004911	0.038737	1021|5581|4915|23328|5795|3984|2263|11315|168667|5683
364	response to lipopolysaccharide	GO:0032496	0.004922	0.038737	10068|5581|1543|23328
365	protein modification by small protein conjugation or removal	GO:0070647	0.004968	0.038919	5581|83593|51343|23328|3984|11315|5683
366	cellular protein localization	GO:0034613	0.004972	0.038919	3782|83593|375346|11252|5192|11336|11315|112755|54535
367	immune response-regulating cell surface receptor signaling pathway	GO:0002768	0.005082	0.039675	3984|5795|5683|5581
368	cardiac septum morphogenesis	GO:0060411	0.005116	0.039836	2263|2702
369	cellular macromolecule localization	GO:0070727	0.005139	0.039904	3782|83593|375346|11252|5192|11336|11315|112755|54535

370	regulation of neurotransmitter secretion	GO:0046928	0.005314	0.040827	4915|112755
371	oxygen binding	GO:0019825	0.005314	0.040827	1545|1543
372	lipopolysaccharide-mediated signaling pathway	GO:0031663	0.005314	0.040827	5581|23328
373	eye development	GO:0001654	0.005357	0.040957	1545|51343|1543|4915
374	transmembrane transport	GO:0055085	0.005363	0.040957	3782|5581|375346|6506|2702|148808|11315|5192
375	regulation of epithelial cell migration	GO:0010632	0.005373	0.040957	5581|168667|23328
376	artery morphogenesis	GO:0048844	0.005515	0.041873	5396|2702
377	regulation of transcription, DNA-templated	GO:0006355	0.005541	0.041873	2120|1545|57491|5396|7716|7799|389058|55657|2005|7553|2263|11315|168667|22887|1893
378	appendage development	GO:0048736	0.00555	0.041873	2263|5396|2702
379	limb development	GO:0060173	0.00555	0.041873	2263|5396|2702
380	response to molecule of bacterial origin	GO:0002237	0.005699	0.042812	10068|5581|1543|23328
381	positive regulation of phospholipase activity	GO:0010518	0.005719	0.042812	2263|5581
382	arachidonic acid metabolic process	GO:0019369	0.005719	0.042812	1545|1543
383	neuron projection morphogenesis	GO:0048812	0.005736	0.042826	3984|91653|4915|127833|2263
384	protein kinase binding	GO:0019901	0.005779	0.043041	5829|5795|112755|4915|23328
385	regulation of biosynthetic process	GO:0009889	0.005801	0.043092	2120|1545|57491|5396|7716|2208|10643|7799|389058|55657|2005|7553|2263|11315|168667|22887|1893
386	regulation of nucleic acid-	GO:1903506	0.005821	0.043135	2120|1545|57491|5396|7716|7799|389058|55657|2005|7553|2263|11315|168667|22887|1893

	templated transcription				
387	inner ear development	GO:0048839	0.005916	0.043645	2263|168667|5396
388	regulation of cellular macromolecule biosynthetic process	GO:2000112	0.005921	0.043645	2120|1545|57491|5396|7716|10643|7799|389058|55657|2005|7553|2263|11315|168667|22887|1893
389	epithelial cell proliferation	GO:0050673	0.005935	0.043645	2263|1021|168667|1893
390	behavior	GO:0007610	0.006001	0.044017	11315|6506|4915|89797|5581
391	plasma membrane part	GO:0044459	0.00603	0.044116	91653|2208|4915|11252|6506|2702|3588|5795|59352|2838|2263|5799
392	regulation of RNA biosynthetic process	GO:2001141	0.006097	0.044441	2120|1545|57491|5396|7716|7799|389058|55657|2005|7553|2263|11315|168667|22887|1893
393	regulation of cell-substrate adhesion	GO:0010810	0.006105	0.044441	5795|1021|5581
394	ventricular septum development	GO:0003281	0.006138	0.044575	2263|2702
395	generation of neurons	GO:0048699	0.00622	0.045053	91653|1021|5396|57465|127833|4915|3984|2263
396	positive regulation of cytoskeleton organization	GO:0051495	0.006296	0.045496	3984|10788|5581
397	tetrapyrrole metabolic process	GO:0033013	0.006353	0.045791	1371|1543
398	regulation of ossification	GO:0030278	0.006394	0.04597	2263|1021|1893
399	regulation of nitrogen compound metabolic process	GO:0051171	0.006546	0.046783	2120|1545|57491|5396|7716|10643|7799|389058|55657|22887|2005|7553|2263|11315|168667|5683|1893
400	insulin secretion involved in cellular response to glucose stimulus	GO:0035773	0.006571	0.046783	5799|5581
401	cranial skeletal system	GO:1904888	0.006571	0.046783	2263|5396

	development				
402	cartilage development	GO:0051216	0.006591	0.04681	100526772|1297|5396
403	positive regulation of protein ubiquitination	GO:0031398	0.006691	0.047343	83593|51343|5683
404	heterocycle biosynthetic process	GO:0018130	0.006698	0.047343	2120|1284|57491|5396|1545|7716|7799|1371|389058|55657|2005|7553|2263|11315|168667|22887|1893
405	regulation of protein complex assembly	GO:0043254	0.006748	0.047577	11315|10788|112755|5581
406	secondary metabolic process	GO:0019748	0.006792	0.047661	1545|1543
407	regulation of calcium ion-dependent exocytosis	GO:0017158	0.006792	0.047661	127833|112755
408	developmental growth	GO:0048589	0.006849	0.04794	3984|2263|100526772|6506|127833
409	regulation of transmembrane transporter activity	GO:0022898	0.006893	0.04807	375346|11315|5581
410	aromatic compound biosynthetic process	GO:0019438	0.0069	0.04807	2120|1284|57491|5396|1545|7716|7799|1371|389058|55657|2005|7553|2263|11315|168667|22887|1893
411	protein export from nucleus	GO:0006611	0.007017	0.048652	11315|54535
412	establishment of localization in cell	GO:0051649	0.007126	0.04929	3782|5581|127833|375346|4915|5192|11315|112755|54535|5799
413	forebrain development	GO:0030900	0.007146	0.049294	2263|4915|1021|6506
414	Fc receptor signaling pathway	GO:0038093	0.007204	0.049294	3984|5683|5581
415	locomotory behavior	GO:0007626	0.007204	0.049294	11315|89797|5581
416	outflow tract morphogenesis	GO:0003151	0.007245	0.049294	2263|2702
417	exocytic vesicle membrane	GO:0099501	0.007245	0.049294	127833|5799
418	regulation of	GO:0032768	0.007245	0.049294	2208|11315

	monooxygenase activity				
419	synaptic vesicle membrane	GO:0030672	0.007245	0.049294	127833|5799
420	positive regulation of organelle organization	GO:0010638	0.007354	0.049919	3782|3984|11315|10788|5581

Table S4. The relationship between methylation and expression

	Probe	Chromosome	Gene_Symbol	The relationship between probe and expression	Correlation coefficient
1	cg00976097	chr5	AHRR	Positive	0.22
2	cg05575921	chr5	AHRR	Negative	-0.13
3	cg26076054	chr5	AHRR	Positive	0.21
4	cg13990746	chr1	ANKRD45	Negative	-0.16
5	cg15680620	chr1	ANKRD45	Negative	-0.13
6	cg09799983	chr2	CYP1B1	Negative	-0.11
7	cg11751707	chr2	CYP1B1	Positive	0.00
8	cg09194449	chr7	PTPRN2	Negative	-0.08
9	cg10650290	chr7	PTPRN2	Positive	0.14
10	cg14419740	chr7	PTPRN2	Negative	-0.14
11	cg07813142	chr2	SP5	Negative	-0.07
12	cg24772753	chr2	SP5	Negative	-0.05
13	cg05752786	chr1	SYT2	Positive	0.01
14	cg16315376	chr1	SYT2	Negative	-0.01
15	cg26582784	chr7	AC002454.1	Positive	0.03
16	cg00993400	chr1	AL672294.1	Positive	0.02
17	cg07325233	chr21	AP000295.9	Positive	0.16
18	cg02826525	chr2	ARHGEF33	Negative	-0.10
19	cg21500300	chr12	BCAT1	Negative	-0.13
20	cg10413224	chr7	BMPER	Positive	0.13
21	cg02498206	chr3	BOC	Positive	0.08
22	cg25192619	chr6	CCDC167	Negative	-0.07
23	cg00688979	chr6	CCHCR1	Positive	0.07
24	cg02050426	chr18	CDH20	Positive	0.06
25	cg17373442	chr3	CHST2	Negative	-0.06
26	cg04616529	chr16	CLEC16A	Negative	-0.09
27	cg10076730	chr13	COL4A2	Positive	0.00
28	cg15922705	chr6	COL9A1	Positive	0.21
29	cg08374798	chr20	COL9A3	Positive	0.03
30	cg19859270	chr3	CPOX	Negative	-0.10
31	cg00370022	chr15	CYP1A1	Negative	-0.14
32	cg17211612	chr12	DNAH10	Positive	0.01
33	cg05951221	chr2	ECEL1P1	Negative	-0.05
34	cg26799398	chr1	ECM1	Negative	-0.32
35	cg21012061	chr1	ELK4	Negative	-0.12
36	cg24279017	chr12	ETV6	Positive	0.07
37	cg16322479	chr5	EXOC3	Negative	-0.05
38	cg26005485	chr8	FAM135B	Positive	0.07
39	cg01637537	chr22	FAM83F	Positive	0.07
40	cg12387247	chr19	FCER2	Negative	-0.03

41	cg05033369	chr1	FCRLA	Positive	0.01
42	cg01049916	chr10	FGFR2	Positive	0.32
43	cg09278187	chr1	FOXJ3	Negative	-0.03
44	cg13563863	chr19	FZR1	Negative	-0.05
45	cg08733957	chr1	GALE	Negative	-0.06
46	cg08894131	chr1	GJA5	Negative	-0.13
47	cg03078488	chr7	IGF2BP3	Negative	-0.10
48	cg20538211	chr4	IGFBP7-AS1	Negative	-0.07
49	cg26972614	chr11	IL18BP	Negative	-0.03
50	cg02387679	chr5	IQGAP2	Negative	-0.21
51	cg18713316	chr1	KCNN3	Negative	-0.01
52	cg00702638	chr3	KIAA1143	Negative	-0.05
53	cg03985801	chr1	LGR6	Positive	0.17
54	cg00334821	chr7	LIMK1	Negative	0.00
55	cg03277049	chr3	LINC00886	Positive	0.00
56	cg16377959	chr5	LINC01019	Negative	-0.12
57	cg10354195	chr10	LRRC27	Positive	0.08
58	cg15513657	chr14	MEG3	Positive	0.06
59	cg18001059	chr7	MRPL32	Negative	0.00
60	cg24203542	chr11	NAV2	Negative	-0.09
61	cg13654445	chr9	NTRK2	Positive	0.11
62	cg18919659	chr22	PACSIN2	Negative	-0.08
63	cg21885107	chr14	PAPLN	Negative	-0.03
64	cg10385390	chr1	PARK7	Positive	0.01
65	cg14373988	chr1	PEX10	Negative	-0.12
66	cg03945895	chr1	PRDM2	Positive	0.00
67	cg16884847	chr2	PRKCE	Negative	-0.13
68	cg11954332	chr1	PRRX1	Positive	0.09
69	cg11545521	chr11	PTPRJ	Negative	-0.11
70	cg23854567	chr12	PXN	Positive	0.02
71	cg16840978	chr8	RAB11FIP1	Negative	-0.03
72	cg15585555	chr1	RASSF5	Negative	-0.23
73	cg08008475	chr13	RNY1P1	Positive	0.18
74	cg09370982	chr16	RP11-20I23.1	Positive	0.16
75	cg20628376	chr10	RP11-351M16.3	Negative	-0.13
76	cg07709148	chr8	RP11-486M23.1	Negative	-0.04
77	cg14270346	chr9	RP11-613M10.9	Negative	-0.05
78	cg23501962	chr11	RP4-683L5.1	Positive	0.03
79	cg23369748	chr6	SASH1	Positive	0.04
80	cg15233380	chr13	SHISA2	Positive	0.25
81	cg05787209	chr16	STX1B	Negative	-0.14
82	cg03642695	chr17	TEKT3	Positive	0.07
83	cg04865290	chr3	TMEM110	Negative	-0.13
84	cg20439473	chr17	VEZF1	Negative	-0.07

85	cg26029292	chr8	ZNF7	Negative	-0.04

Note: The remaining 20 probes do not correspond to the gene.

Table S5. The expression of 95 genes corresponding to 105 probes (current-smoker VS never-smoker)

	Gene_Symbol	Chromosome	Start	End	Location	Type
1	AC002454.1	7	92836483	92917187	7q21.2	Over-expression
2	AHRR	5	304176	438291	5p15.33	Over-expression
3	AL672294.1	1	2.49E+08	2.49E+08	1q44	Under-expression
4	ANKRD45	1	1.74E+08	1.74E+08	1q25.1	Under-expression
5	AP000295.9	21	33246774	33283212	21q22.11	Under-expression
6	ARHGEF33	2	38889880	38975449	2p22.1	Under-expression
7	BCAT1	12	24810022	24949459	12p12.1	Under-expression
8	BMPER	7	33904911	34155872	7p14.3	Under-expression
9	BOC	3	1.13E+08	1.13E+08	3q13.2	Under-expression
10	CCDC167	6	37482920	37499922	6p21.2	Over-expression
11	CCHCR1	6	31142439	31158238	6p21.33	Over-expression
12	CDH20	18	61333582	61555773	18q21.33	Under-expression
13	CDK6	7	92604921	92836594	7q21.2	Over-expression
14	CHST2	3	1.43E+08	1.43E+08	3q24	Under-expression
15	CLEC16A	16	10944488	11182189	16p13.13	Under-expression
16	COL4A2	13	1.1E+08	1.11E+08	13q34	Over-expression
17	COL9A1	6	70215061	70303083	6q13	Under-expression
18	COL9A3	20	62816244	62841159	20q13.33	Under-expression
19	CPOX	3	98521132	98593723	3q11.2	Over-expression
20	CYP1A1	15	74719542	74725610	15q24.1	Over-expression
21	CYP1B1	2	38066973	38109902	2p22.2	Under-expression
22	CYP1B1-AS1	2	38073447	38231651	2p22.2	Under-expression
23	DNAH10	12	1.24E+08	1.24E+08	12q24.31	Under-expression
24	ECEL1P1	2	2.32E+08	2.32E+08	2q37.1	Over-expression
25	ECM1	1	1.51E+08	1.51E+08	1q21.2	Under-expression
26	ELK4	1	2.06E+08	2.06E+08	1q32.1	Under-expression
27	ETV6	12	11649854	11895402	12p13.2	Over-expression
28	EXOC3	5	443158	471937	5p15.33	Over-expression
29	EXOC3-AS1	5	441498	443160	5p15.33	Over-expression
30	FAM135B	8	1.38E+08	1.38E+08	8q24.23	Under-expression
31	FAM83F	22	39994949	40043529	22q13.1	Over-expression
32	FCER2	19	7688758	7702146	19p13.2	Under-expression
33	FCRLA	1	1.62E+08	1.62E+08	1q23.3	Over-expression
34	FGFR2	10	1.21E+08	1.22E+08	10q26.13	Under-expression
35	FOXJ3	1	42176539	42335877	1p34.2	Over-expression
36	FZR1	19	3506273	3538330	19p13.3	Over-expression
37	GALE	1	23795599	23800804	1p36.11	Under-expression
38	GJA5	1	1.48E+08	1.48E+08	1q21.2	Under-expression

39	GPR15	3	98531899	98533150	3q11.2	Over-expression
40	IGF2BP3	7	23310209	23470467	7p15.3	Over-expression
41	IGFBP7-AS1	4	57109762	57205510	4q12	Under-expression
42	IL10RB	21	33266358	33297234	21q22.11	Under-expression
43	IL10RB-AS1	21	33263873	33266260	21q22.11	Under-expression
44	IL18BP	11	71998541	72005715	11q13.4	Over-expression
45	IQGAP2	5	76403249	76708132	5q13.3	Under-expression
46	KCNN3	1	1.55E+08	1.55E+08	1q21.3	Under-expression
47	KIAA1143	3	44737661	44761662	3p21.31	Under-expression
48	KIF15	3	44761717	44873376	3p21.31	Over-expression
49	LGR6	1	2.02E+08	2.02E+08	1q32.1	Under-expression
50	LIMK1	7	74082933	74122525	7q11.23	Under-expression
51	LINC00886	3	1.57E+08	1.57E+08	3q25.31	Under-expression
52	LINC01019	5	3417152	3536094	5p15.33	Over-expression
53	LRRC27	10	1.32E+08	1.32E+08	10q26.3	Under-expression
54	MEG3	14	1.01E+08	1.01E+08	14q32.2	Under-expression
55	MFSD4	1	2.06E+08	2.06E+08	1q32.1	Under-expression
56	MRPL32	7	42932200	42948958	7p14.1	Under-expression
57	NAV2	11	19350724	20121598	11p15.1	Over-expression
58	NTRK2	9	84668551	85027070	9q21.33	Under-expression
59	PACSIN2	22	42835412	43015145	22q13.2	Under-expression
60	PAPLN	14	73237497	73274640	14q24.2	Under-expression
61	PARK7	1	7954291	7985505	1p36.23	Under-expression
62	PEX10	1	2403964	2413797	1p36.32	Over-expression
63	PRDM2	1	13700198	13825079	1p36.21	Under-expression
64	PRKCE	2	45651345	46187990	2p21	Under-expression
65	PRRX1	1	1.71E+08	1.71E+08	1q24.2	Under-expression
66	PSMA2	7	42916857	42932223	7p14.1	Under-expression
67	PTPRJ	11	47980558	48170841	11p11.2	Under-expression
68	PTPRN2	7	1.58E+08	1.59E+08	7q36.3	Under-expression
69	PXN	12	1.2E+08	1.2E+08	12q24.23	Over-expression
70	RAB11FIP1	8	37858618	37899467	8p11.23	Under-expression
71	RASSF5	1	2.07E+08	2.07E+08	1q32.1	Under-expression
72	RNY1P1	13	27402476	27402587	13q12.2	Over-expression
73	RP11-173C1.1	2	38959287	38960342	2p22.1	Over-expression
74	RP11-20I23.1	16	2496032	2520218	16p13.3	Over-expression
75	RP11-351M16.3	10	28433008	28495813	10p12.1	Over-expression
76	RP11-486M23.1	8	29527312	29530323	8p12	Over-expression
77	RP11-613M10.9	9	37588413	38068687	9p13.2	Under-expression
78	RP11-662I13.2	12	24949163	24960158	12p12.1	Over-expression
79	RP4-647C14.2	14	73242651	73245979	14q24.2	Over-expression
80	RP4-683L5.1	11	35419057	35421002	11p13	Over-expression
81	SASH1	6	1.48E+08	1.49E+08	6q24.3	Under-expression
82	SHB	9	37919134	38069211	9p13.1	Under-expression

83	SHISA2	13	26044597	26051031	13q12.13	Under-expression
84	SLC1A2	11	35251206	35420063	11p13	Under-expression
85	SP5	2	1.71E+08	1.71E+08	2q31.1	Over-expression
86	STX1B	16	30989256	31010628	16p11.2	Under-expression
87	SYT2	1	2.03E+08	2.03E+08	1q32.1	Under-expression
88	TARS2	1	1.5E+08	1.51E+08	1q21.2	Over-expression
89	TBC1D24	16	2475118	2505734	16p13.3	Under-expression
90	TEKT3	17	15303811	15341641	17p12	Under-expression
91	TMEM110	3	52836219	52897596	3p21.1	Over-expression
92	TMEM110-MUSTN1	3	52833121	52897562	3p21.1	Over-expression
93	VEZF1	17	57971547	57988259	17q22	Under-expression
94	ZNF692	1	2.49E+08	2.49E+08	1q44	Over-expression
95	ZNF7	8	1.45E+08	1.45E+08	8q24.3	Over-expression

Table S6. Related references and related types of 95 genes

	Gene	Related types	Related references or materials
1	AC002454.1	cancer	https://www.genecards.org/cgi-bin/carddisp.pl?gene=LOC101927497
2	AHRR	smoking; lung cancer	Cigarette smoking and DNA methylation; Hypomethylation of smoking-related genes is associated with future lung cancer in four prospective cohorts
3	ANKRD45	cancer	Ankrd45 Is a Novel Ankyrin Repeat Protein Required for Cell Proliferation
4	CCDC167	cancer	https://www.proteinatlas.org/ENSG00000198937-CCDC167/pathology
5	CCHCR1	cancer	Centrosomal Localization of the Psoriasis Candidate Gene Product, CCHCR1, Supports a Role in Cytoskeletal Organization
6	CDK6	smoking	Cigarette tar phenols impede T cell cycle progression by inhibiting cyclin-dependent kinases
7	CHST2	smoking	Differential gene expression patterns between smokers and non-smokers: cause or consequence?
8	CLEC16A	cancer	https://www.proteinatlas.org/ENSG00000038532-CLEC16A/pathology
9	COL4A2	cancer	siRNA-Mediated suppression of collagen type iv alpha 2 (COL4A2) mRNA inhibits triple-negative breast cancer cell proliferation and migration
10	COL9A1	smoking	Variants in FAT1 and COL9A1 genes in male population with or without substance use to assess the risk factors for oral malignancy
11	COL9A3	cancer	Genomic Deregulation during Renal Cell Carcinoma Metastasis Implements a Myofibroblast-Like Gene Expression Program
12	CPOX	smoking	CpG Sites Associated with Cigarette Smoking: Analysis of Epigenome-Wide Data from the Sister Study
13	CYP1A1	cancer	Association between cytochrome P450 1A1 (CYP1A1) gene polymorphisms and the risk of renal cell carcinoma: a meta-analysis
14	CYP1B1	smoking; lung cancer	Interaction of CYP1B1, cigarette-smoke carcinogen metabolism, and lung cancer risk
15	CYP1B1-AS1	smoking	A combined epigenome- and transcriptome-wide association study of the oral masticatory mucosa assigns CYP1B1 a central role for epithelial health in smokers

16	DNAH10	smoking	FOXJ1 Prevents Cilia Growth Inhibition by Cigarette Smoke in Human Airway Epithelium In Vitro
17	ECM1	smoking	Identification of novel target genes in human lung tissue involved in chronic obstructive pulmonary disease
18	ELK4	NSCLC	Resection of Non–Small Cell Lung Cancers Reverses Tumor-Induced Gene Expression Changes in the Peripheral Immune System
19	ETV6	smoking; cancer	Correlates of prenatal and early-life tobacco smoke exposure and frequency of common gene deletions in childhood acute lymphoblastic leukemia; Common genetic variation in ETV6 is associated with colorectal cancer susceptibility
20	EXOC3	smoking	Smoking induces DNA methylation changes in Multiple Sclerosis patients with exposure-response relationship
21	EXOC3-AS1	NSCLC	LncRNAs are altered in lung squamous cell carcinoma and lung adenocarcinoma
22	FAM135B	lung cancer	Comprehensive genomic profiling of small cell lung cancer in Chinese patients and the implications for therapeutic potential
23	FAM83F	luad	FAM83 family oncogenes are broadly involved in human cancers: an integrative multi-omics approach
24	FCER2	lung cancer	Polymorphisms in innate immunity genes and lung cancer risk in Xuanwei, China
25	FCRLA	smoking	Maternal smoking impacts key biological pathways in newborns through epigenetic modification in Utero
26	FGFR2	smoking; cancer	FGFR2 intronic SNPs and breast cancer risk: associations with tumor characteristics and interactions with exogenous exposures and other known breast cancer risk factors
27	FOXJ3	lung cancer	MiR-517a-3p accelerates lung cancer cell proliferation and invasion through inhibiting FOXJ3 expression
28	GPR15	smoking	Novel DNA Methylation Sites Influence GPR15 Expression in Relation to Smoking
29	IGF2BP3	smoking; lung cancer	Effects of Cigarette Smoke on the Human Oral Mucosal Transcriptome; Insulin-like growth factor 2 mRNA binding protein 3 (IGF2BP3) promotes lung tumorigenesis via attenuating p53 stability
30	IL10RB	smoking	Axonal guidance signaling pathway interacting with smoking in modifying the risk of pancreatic cancer: a gene- and pathway-based interaction analysis of GWAS data
31	IL10RB-AS1	cancer	Benchmark of lncRNA Quantification for RNA-Seq of Cancer Samples
32	IQGAP2	cancer	Reduced expression of IQGAP2 and higher expression of IQGAP3 correlates with poor prognosis in cancers
33	KCNN3	smoking	KCNN Genes that Encode Small-Conductance Ca2+-Activated K+ Channels Influence Alcohol and Drug Addiction
34	KIAA1143	smoking; luad	Meta-analysis of mRNA expression profiles to identify differentially expressed genes in lung adenocarcinoma tissue from smokers and non-smokers
35	KIF15	luad	Increased KIF15 Expression Predicts a Poor Prognosis in Patients with Lung Adenocarcinoma
36	LGR6	smoking	Differential gene expression patterns between smokers and non-smokers: cause or consequence?
37	LIMK1	smoking; cancer	PI3K Catalytic Isoform Alteration Promotes the LIMK1-related Metastasis Through the PAK1 or ROCK1/2 Activation in Cigarette Smoke-exposed Ovarian Cancer Cells
38	LINC00886	lusc	LncRNAs are altered in lung squamous cell carcinoma and lung adenocarcinoma
39	LINC01019	smoking	DNA methylation and smoking in Korean adults: epigenome-wide association study

40	MEG3	lung cancer	Association Between Long Noncoding RNA MEG3 Polymorphisms and Lung Cancer Susceptibility in Chinese Northeast Population
41	MFSD4	cancer	Metastatic pathway-specific transcriptome analysis identifies MFSD4 as a putative tumor suppressor and biomarker for hepatic metastasis in patients with gastric cancer
42	NAV2	cancer	Neuron navigator 2 overexpression indicates poor prognosis of colorectal cancer and promotes invasion through the SSH1L/cofilin-1 pathway
43	NTRK2	smoking	Gene-gene interactions among CHRNA4, CHRNB2, BDNF and NTRK2 in nicotine dependence
44	PACSIN2	smoking	Gene Expression Signature of Cigarette Smoking and Its Role in Lung Adenocarcinoma Development and Survival
45	PARK7	NSCLC	Status of the Parkinson's disease gene family expression in non-small-cell lung cancer
46	PRDM2	NSCLC	The methylation profiles of PRDM promoters in non–small cell lung cancer
47	PRRX1	cancer	Aberrant expression of vasculogenic mimicry, PRRX1, and CIP2A in clear cell renal cell carcinoma and its clinicopathological significance
48	PSMA2	lung cancer	The transcription levels and prognostic values of seven proteasome alpha subunits in human cancers
49	PTPRJ	lung cancer	The receptor protein tyrosine phosphatase PTPRJ negatively modulates the CD98hc oncoprotein in lung cancer cells
50	PTPRN2	luad	Frequent silencing of the candidate tumor suppressor TRIM58 by promoter methylation in early-stage lung adenocarcinoma
51	PXN	cancer	Paxillin promotes colorectal tumor invasion and poor patient outcomes via ERK-mediated stabilization of Bcl-2 protein by phosphorylation at Serine 87
52	RAB11FIP1	cancer	RCP is a human breast cancer-promoting gene with Ras-activating function
53	RASSF5	cancer	Frequent promoter hypermethylation of tumor-related genes in head and neck squamous cell carcinoma
54	SASH1	smoking	SASH1, a new potential link between smoking and atherosclerosis
55	SHISA2	cancer	SHISA2 enhances the aggressive phenotype in prostate cancer through the regulation of WNT5A expression
56	SLC1A2	smoking	Glutamate and Synaptic Plasticity Systems and Smoking Behavior: Results from a Genetic Association Study
57	TBC1D24	cancer	Downregulation of TBC1 Domain Family Member 24 (BC1D24) Inhibits Breast Carcinoma Growth via IGF1R/PI3K/AKT Pathway
58	TEKT3	lusc	Weighted gene co-expression network analysis in identification of metastasis-related genes of lung squamous cell carcinoma based on the Cancer Genome Atlas database
59	TMEM110-MUSTN1	luad	Identification of Proteomic Features To Distinguish Benign Pulmonary Nodules from Lung Adenocarcinoma
60	ZNF692	luad	ZNF692 promotes proliferation and cell mobility in lung adenocarcinoma
61	ZNF7	cancer	https://www.proteinatlas.org/ENSG00000147789-ZNF7/pathology

Note: The remaining 34 genes involved in the disease are not yet clear. luad: lung adenocarcinoma; lusc: lung squamous cell carcinoma; NSCLC: Non-small cell lung cancer

Table S7. The 33 possible related genes corresponding to proteins whose betweenness greater than or equal to 100 obtained by BIMC method using the shortest path algorithm and PPI network

	hgnc_symbol	Ensembl	Betweenness

1	MAGOH	ENSP00000360525	289
2	FYN	ENSP00000346671	204
3	CBL	ENSP00000264033	162
4	RHOA	ENSP00000400175	158
5	MAPK8	ENSP00000353483	157
6	CYC1	ENSP00000317159	152
7	ITGB1	ENSP00000303351	150
8	CCL26	ENSP00000005180	149
9	CYCS	ENSP00000307786	149
10	DHX8	ENSP00000262415	148
11	CCR1	ENSP00000296140	148
12	CDC40	ENSP00000304370	148
13	PRPF18	ENSP00000367835	148
14	CCR5	ENSP00000292303	144
15	WDR5	ENSP00000351446	143
16	CXCR4	ENSP00000386884	142
17	CFTR	ENSP00000003084	142
18	GSTP1	ENSP00000381607	140
19	MT-ATP6	ENSP00000354632	139
20	MT-CO3	ENSP00000354982	139
21	ATP5F1A	ENSP00000282050	139
22	OGT	ENSP00000362824	138
23	HCFC1	ENSP00000309555	138
24	PPP1CB	ENSP00000296122	138
25	ITGA4	ENSP00000380227	124
26	PXN	ENSP00000228307	123
27	SDC2	ENSP00000307046	118
28	FGF2	ENSP00000264498	116
29	FGFR1	ENSP00000393312	116
30	SERPINC1	ENSP00000356671	113
31	KCTD10	ENSP00000228495	106
32	ATXN3	ENSP00000376965	106
33	RPS3	ENSP00000433821	100

Supplementary References

Abdi, H. and Williams, L.J. Partial least squares methods: partial least squares correlation and partial least square regression. *Methods Mol Biol* 2013;930:549-579.
Nguyen, D.V. and Rocke, D.M. Tumor classification by partial least squares using microarray gene expression data. *Bioinformatics* 2002;18(1):39-50.
Qiu, Z.W.*, et al.* Genome-wide copy number variation pattern analysis and a classification signature for non-small cell lung cancer. *Genes Chromosomes Cancer* 2017;56(7):559-569.
Song, K.*, et al.* Classifier assessment and feature selection for recognizing short coding sequences of human genes. *J Comput Biol* 2012;19(3):251-260.
Yuan, L.*, et al.* Integration of Multi-omics Data for Gene Regulatory Network Inference and Application to Breast Cancer. *IEEE/ACM Trans Comput Biol Bioinform* 2018.
